# Effectiveness of a coordinated support system linking public hospitals to a health coaching service compared with usual care at discharge for patients with chronic low back pain: protocol for a randomised controlled trial

**DOI:** 10.1186/s12891-021-04479-z

**Published:** 2021-07-09

**Authors:** Emma K. Ho, Manuela L. Ferreira, Adrian Bauman, Paul W. Hodges, Christopher G. Maher, Milena Simic, Rachael L. Morton, Chris Lonsdale, Qiang Li, Melissa T. Baysari, Anita B. Amorim, Dragana Ceprnja, Ornella Clavisi, Mark Halliday, Matthew Jennings, Alice Kongsted, Katherine Maka, Kate Reid, Tahlia Reynolds, Paulo H. Ferreira

**Affiliations:** 1 The University of Sydney, Faculty of Medicine and Health, Sydney, NSW Australia; 2 The University of Queensland, School of Health and Rehabilitation Sciences, Brisbane, QLD Australia; 3 Institute for Musculoskeletal Health, The University of Sydney and Sydney Local Health District, Sydney, NSW Australia; 4 NHMRC Clinical Trials Centre, Faculty of Medicine and Health, The University of Sydney, Camperdown, NSW Australia; 5 Faculty of Health Sciences, Australian Catholic University, North Sydney, NSW Australia; 6 The George Institute for Global Health, University of New South Wales, Sydney, Australia; 7 Discipline of Biomedical Informatics and Digital Health, School of Medical Sciences, Charles Perkins Centre, Faculty of Medicine and Health, The University of Sydney, Sydney, NSW Australia; 8 Physiotherapy Department, Westmead Hospital, Sydney, NSW Australia; 9 Musculoskeletal Australia, Muscle Bone & Joint Health Ltd, Melbourne, VIC Australia; 10 Concord Repatriation General Hospital, Sydney, NSW Australia; 11 South Western Sydney Local Health District, Sydney, NSW Australia; 12 Department of Sports Sciences and Clinical Biomechanics, University of Southern Denmark, Odense, Denmark; 13 The Centre for Population Health, NSW Ministry of Health, Sydney, NSW Australia

**Keywords:** Chronic low back pain, Randomised controlled trial, Health coaching

## Abstract

**Background:**

Although many people with chronic low back pain (LBP) improve following conservative treatment, one in five will experience worsening symptoms after discharge from treatment and seek health care again. The current LBP clinical care pathway in many health services lacks a well-integrated, systematic approach to support patients to remain physically active and self-manage their symptoms following discharge from treatment. Health coaching can support people to improve physical activity levels and may potentially reduce health care utilisation for LBP. The primary aim of this study is to evaluate the effect of introducing a coordinated support system (linking hospital outpatient physiotherapy services to a public health coaching service) at discharge from LBP treatment, on the future use of hospital, medical, and health services for LBP, compared with usual care provided at discharge.

**Methods:**

Three hundred and seventy-four adults with chronic non-specific LBP will be recruited from the outpatient physiotherapy departments of public hospitals in New South Wales, Australia. Participants will be individually randomised to a support system (*n* = 187) or usual care group (*n* = 187). All participants will receive usual care provided at discharge from treatment. Participants allocated to the support system will also receive up to 10 telephone-based health coaching sessions, delivered by the Get Healthy Service®, over a 6-month period. Health coaches will monitor and support participants to improve physical activity levels and achieve personal health-related goals. The primary outcome is the total number of encounters with hospital, medical, and health services for LBP, at 12 months from baseline. A within-trial economic evaluation will quantify the incremental costs and benefits of the support system from a health system perspective, to support reimbursement decision making.

**Discussion:**

This study will establish the effect of a coordinated support system, introduced at discharge from treatment, on the future use of hospital, medical, and health services for LBP and various health outcomes.

**Conclusion:**

Innovative community-driven solutions to support people with chronic LBP after discharge from treatment are urgently needed. Study findings will help inform health care policy and clinical practice in Australia.

**Trial Registration:**

Prospectively registered on the Australian New Zealand Clinical Trials Registry (ACTRN12620000889954) on 10/09/2020.

**Supplementary Information:**

The online version contains supplementary material available at 10.1186/s12891-021-04479-z.

## Background

Low back pain (LBP) is the leading contributor to disability in Australia and globally [1, 2]. In Australia, nearly 4 million individuals report LBP at any one time [3] and the total cost of treatment exceeds $9 billion annually [4]. Within the current model of care for LBP in most countries, patients are commonly referred to primary care for treatment, including physiotherapy services (private clinics and hospital outpatient departments) [5, 6]. Although many people with LBP continue to improve following discharge from treatment, with approximately one third of individuals recovering within the first 9 months [7], one in five people experience recurrence of pain [8, 9] and resort to seeking further care within 12 months [10, 11]. For patients discharged after receiving treatment for LBP from hospital outpatient physiotherapy departments, this extra care may include additional pain medication intake, re-entry to the hospital system for further outpatient physiotherapy treatment, presentation to the emergency department, or surgical intervention.

In Australia, local hospital networks are responsible for managing and linking public hospitals, health institutions, and health services across defined geographical areas [12]. This includes the provision of hospital-based outpatient physiotherapy services for people with LBP. In the Australian state of New South Wales, 15 local hospital networks (called local health districts) service a total population of 8.2 million people, across eight metropolitan and seven rural and regional locations. In the Western Sydney Local Health District, an ethnically and culturally diverse metropolitan region, the rate of re-presentation to hospital services (i.e., physiotherapy clinics, emergency departments, pain clinics, neurosurgical clinics) within 1 year after discharge from outpatient physiotherapy treatment for LBP is 21% (unpublished New South Wales hospital data). The high rate of re-presentation constitutes a financial burden of $AUD744,000 yearly in direct costs in this local health district alone. Extrapolating these estimates across all local health districts within New South Wales, the cumulative financial and resource burden of re-presentations following discharge from hospital outpatient physiotherapy treatment is undoubtedly substantial.

The decline in clinical outcomes and the additional use of care (i.e., hospital, medical, and health services) for this subset of the LBP population is likely to be amplified by the lack of an integrated, systematic, local health district-driven approach to support patients to self-manage their condition once physiotherapy treatment ceases. After a series of consultations with senior musculoskeletal clinicians and consumer groups representing patients with LBP in Sydney, Australia, we identified that the lack of a coordinated support system at discharge is considered a strong factor driving the pattern of patients returning to hospital for further treatment (unpublished New South Wales hospital consumer committee report). Patients who participated in focus groups expressed concerns regarding the overload of information delivered abruptly prior to discharge from treatment, as well as the lack of ongoing support available. As a result, patients reported poor confidence for self-management of symptoms and maintenance of positive health behaviours (i.e., adherence to exercise). The integration of a simple, low-cost but well-structured post-discharge support system into the care pathway of chronic LBP is likely to improve outcomes.

Health coaching is a behavioural approach that aims to support individuals living with chronic conditions to adopt sustainable health-promoting behaviours and improve their quality of life [13]. The approach is strongly grounded in evidence-based behaviour-change theories such as Social Influence Theory and the Trans-theoretical Model [14], and typically involves a qualified health coach using motivational interviewing techniques to support patients in achieving collaborative goals and empowering self-management of symptoms [15–17]. Evidence supports that telephone-based health coaching can result in clinically important improvements in physical activity in patients with chronic LBP [18]. This is important because people with chronic LBP who engage in moderate to high-intensity leisure-time physical activity have better outcomes in terms of pain, disability, and quality of life, than those who fail to maintain adequate levels of physical activity [11, 19–21]. Our pilot study of a health coaching intervention for LBP has provided evidence that a telephone-based health coaching intervention is acceptable to LBP patients, can improve physical activity levels, and crucially, may reduce the rate of care-seeking for LBP by 38% [95% confidence interval 0.32 to 1.18] compared with usual care [11]. Thus, health coaching appears to have potential to support people with LBP to remain physically active and reduce their future use of health services for LBP.

The Get Healthy Information and Coaching Service® (Get Healthy Service) delivers a variety of telephone-based health coaching programs for adults with a range of health behavioural risk factors and health complaints in the Australian states of New South Wales, South Australia, and Queensland [22]. Introduced in 2009, this is a well-established and fully operational service, funded by state governments, which provides health coaching programs at no charge for state residents. The goal of the Get Healthy Service® is to improve and support an individual’s capacity to self-manage their own health and wellbeing. The service currently offers a Standard (health) Coaching module which aims to support participants with goal setting, motivation, confidence to overcome barriers, and achievement of sustainable lifestyle changes (i.e., increased physical activity levels, reduced sedentary behaviour). Previous studies have shown that the Get Healthy Service® is effective in improving moderate and vigorous physical activity levels and reducing behavioural risk factors for chronic diseases (i.e., weight, waist circumference, body mass index, nutrition-related behaviours) in the general population [23, 24]. Participants receive up to 10 individually tailored health coaching calls, delivered according to participant preference, over 6 months. The health coaching sessions are led by coaches with university-qualifications in allied health care (i.e., dietetics, exercise physiology), who monitor participants closely throughout the program to ensure they meet their goals safely. The sessions aim to compliment clinical care and offer accountability for treatment plans provided to patients by their clinicians prior to enrolment into the service. The service also has strong clinical governance and appropriate escalation pathways. The Get Healthy Service® is a viable and readily implementable solution that could be systematically integrated in the LBP clinical care pathway, potentially addressing the lack of support available after discharge from treatment across many regions in Australia.

There is only one published randomised controlled trial (RCT) which has evaluated the Get Healthy Service® in people with chronic LBP. The study investigated the effectiveness of a healthy lifestyle intervention (incorporating the Get Healthy Service®) in people with chronic LBP [25], and found no effect on pain intensity, disability, physical activity, or health care use (assessed as health care utilisation over the past 6 weeks preceding assessment) [25]. However, the lack of effect in the study was likely due to poor adherence with treatment, and importantly, the study only recruited overweight or obese patients identified from a waiting list for consultation with an orthopaedic specialist [25]. A protocol paper for a new study has been published for a RCT investigating a healthy lifestyle program involving consultations with a physiotherapist and dietician, provision of educational resources, and referral to the Get Healthy Service and a smoking cessation program [26]. The results of the trial have not been published; however, the protocol describes that study participants will be recruited from a mixed population of patients identified from primary and secondary care, and the general community. Further, the protocol does not describe any measurement of intervention effect on the use of medical or health services for LBP. No studies have investigated the effect of systematically integrating the Get Healthy Service® as a solution to support patients with chronic LBP immediately after discharge from hospital-based care.

This manuscript presents the protocol for a RCT and embedded qualitative study of a coordinated support system introduced after discharge from hospital-based physiotherapy treatment for chronic LBP. The support system will involve a structured referral pathway linking hospital-based outpatient physiotherapy services to a health coaching program, delivered by Get Healthy Service®, which has been tailored for chronic LBP. The primary aim of this study is to evaluate the effect of introducing a coordinated support system at discharge from LBP treatment, on the future use of hospital, medical, and health services for LBP, compared with usual care provided at discharge. The secondary aims of the study are: (i) to investigate the effectiveness and cost-effectiveness of the support system on improving pain, disability, physical activity levels, and quality of life in people with chronic LBP and (ii) to identify factors related to the intervention, context, individual, and implementation process which may contribute to intervention outcomes and to use these findings to inform development of an implementation plan for scalability.

## Methods

### Study design

This is a randomised, single-blind, parallel, superiority clinical trial with 1:1 allocation ratio to either a coordinated support system introduced at discharge from treatment for chronic LBP (involving a structured referral pathway linking hospital-based outpatient physiotherapy services to the Get Healthy Service®), or usual care group (usual care provided at discharge from treatment). We will conduct an embedded qualitative study with key stakeholders, involving a series of in-depth interviews with clinicians and trial participants, and one or more focus groups with health coaches and agents from Get Healthy Service®. The trial protocol has been designed according to the Standard Protocol Items: Recommendations for Interventional Trials (SPIRIT) statement (see Additional file 1) [27]. The study intervention has been reported using the template for intervention description and replication (TIDieR) checklist (see Additional file 2) [28]. The results of the trial will be reported according to the CONsolidated Standards Of Reporting Trials (CONSORT) statement [29]. The trial has been prospectively registered with the Australian New Zealand Clinical Trials Registry (ACTRN12620000889954).

### Participant timeline

**Fig. 1:**
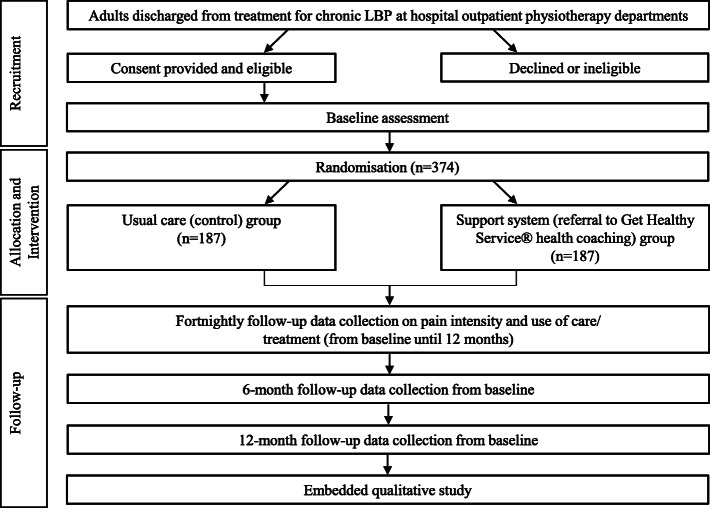
Flow chart of the study

Table 1 shows the assessments at each timepoint following the SPIRIT statement [27]. Figure 1 demonstrates the flow chart of the study.

**Table 1:** Study assessments at specific time points

	STUDY PERIOD					
	Enrolment	Baseline Assessment	Allocation	Follow-up Data Collection^**a**^		
TIMEPOINT*	*Week − 4 to − 1*	*Week 0*	*Week 0*	Fortnightly*	*6 months*	*12 months*
**ENROLMENT**:						
Informed consent	X					
Eligibility screen	X					
Allocation			X			
**INTERVENTIONS**:						
*Support system*			X	X	X	
*Usual care only*			X			
**ASSESSMENTS**:						
*Use of hospital, medical, and health services for LBP*		X		X	X	X
*Self-reported physical activity levels*		X			X	X
*Objective physical activity levels*		X			X	
*Physical Function*		X			X	X
*Pain intensity*		X		X	X	X
*Disability*		X			X	X
*Quality of life*		X			X	X
*Self-management behaviours*		X		X	X	X
*Medication use*		X		X	X	X
*Sleep quality*		X			X	X
*Attitudes regarding use of pain medications*		X			X	X
*Beliefs about back pain*		X			X	X

^a^ From baseline assessment (week 0)

### Participants

We will recruit 374 participants with chronic non-specific LBP from outpatient physiotherapy departments of public hospitals in New South Wales, Australia. Consenting participants will be randomly allocated to either the support system (*n* = 187) or usual care group (*n* = 187).

#### Inclusion criteria

Potential participants will need to meet all the following inclusion criteria:

i) 18 years of age or older;
ii) presentation of non-specific LBP of at least 12-week duration, with or without leg pain but without radicular (e.g., reflex changes, motor loss) symptoms. Non-specific LBP will be defined as LBP without diagnosis of a specific cause, and the absence of serious spinal pathology or indicators of potentially serious conditions using ‘red’ flags;
iii) recently discharged (< 4 weeks post-treatment) from outpatient physiotherapy treatment from a participating hospital site. This includes discharge from one-to-one physiotherapy care directly into the community, or from supervised group exercise programs offered by the outpatient physiotherapy department;
iv) have adequate hearing and eyesight to participate safely in physical activity;
v) independent ambulatory status, with or without a gait aid.

#### Exclusion criteria

Potential participants will be excluded if they have any of the following:

i) known or suspected serious spinal pathology (e.g., fracture, inflammatory disorder); diagnosis of specific LBP (e.g., sciatica, spinal stenosis grade 3 to 4);
ii) co-morbid health condition(s) diagnosed by a medical practitioner that would prevent participation in physical activity or exercise programs;
iii) fibromyalgia or systemic/inflammatory condition; currently pregnant or planning to become pregnant over the study duration;
iv) inadequate English to complete outcome measures or participate in the health coaching intervention;
v) spinal surgery in the past 12 months;
vi) LBP caused by involvement in a road traffic crash in the last 12 months or ongoing compensation.

### Outcomes

#### Primary outcome

The primary outcome will be the total number of encounters with hospital, medical, and health services for LBP (composite measure) [30] over 12 months from baseline assessment. The number of encounters of using hospital, medical, and health services for LBP could be related to a new or ongoing episode of LBP. Data will be collected at baseline, 6- and 12-months from baseline assessment, as well as fortnightly during the 12-month follow-up period, via online (electronic) self-reported questionnaires specifically designed for this study (see Additional file 3, Additional file 4, Additional file 5). Data will also be collected via linkage to participants’ Medicare Benefits Schedule and Pharmaceutical Benefits Scheme data. Encounters with hospital, medical, and health services for LBP will be defined as: (1) a visit to any hospital service due to LBP (e.g., emergency department presentations, inpatient admissions/hospitalisations, outpatient services (e.g., outpatient physiotherapy, pain clinics), surgical procedures due to LBP); (2) a visit to a community-based medical or health practitioner due to LBP (e.g., general practitioner, specialist clinician, physiotherapist); (3) any diagnostic test for LBP (e.g., imaging); (4) a visit to any hospital, medical and health services to receive or fulfill a script for prescription medications, or a visit to any non-hospital Medicare services for LBP. Data on item 4 will be obtained through linkage to participants’ Medicare Benefits Schedule and Pharmaceutical Benefits Scheme data. Each encounter described above will be counted as an individual visit (e.g., a participant receiving 8 sessions of physiotherapy treatment will be considered as having 8 encounters). To account for data dependency, multiple encounters which occur within a 24-h period will be counted as one encounter.

#### Secondary outcomes

The secondary outcomes of this study are described in Table 2. Self-reported data on all secondary outcomes will be collected at baseline, and at 6- and 12-months from baseline assessment, via online (electronic) questionnaires (see Additional file 3, Additional file 4). In addition, self-reported data on pain intensity, self-management behaviours, and medication use will be assessed on a fortnightly basis during the 12-month follow-up period, collected via a repeating online (electronic) questionnaire specifically designed for this study (see Additional file 5).

**Table 2:** Secondary outcomes

Secondary outcome	Measurement tool	Description
Self-reported physical activity levels	Global Physical Activity Questionnaire (GPAQ) [31]	The GPAQ assesses intensity-specific physical activity participation in 3 domains (activity at work, travel to and from places, recreational activities), and sedentary behaviour [31].
Objective physical activity levels	Axivity tri-axial accelerometer [32], assessed over a 7-day period	The device accurately estimates how physically active a person has been throughout the day using an accelerometer. The outcomes are overall physical activity, categorised according to intensity (sedentary, light, moderate, vigorous) and quantified as the average counts per minute of acceleration during the time the accelerometer is worn.
Function	Patient Specific Functional Scale (PSFS) [33]	At baseline, participants will be asked to self-select three activities they have difficulty performing due to their LBP. Each activity will be scored on an 11-point scale at each timepoint, with 0 representing ‘unable to perform activity’ and 10 representing ‘able to perform activity at the same level as before injury or problem.’ The scores for the three activities will be summed, giving a total score ranging from 3 to 30.
Pain intensity (i.e., mean intensity of LBP over the past fortnight)	Numerical Rating Scale (NRS) [34]	The NRS is an 11-point scale, scored on a scale of 0 to 10, with 0 representing ‘no pain’ and 10 representing ‘worst possible pain.’
Disability	Roland–Morris Disability Questionnaire (RMDQ) [35]	The RMDQ consists of 24 items and total scores range from 0 to 24, with higher scores indicating higher disability levels.
Quality of life	Assessment of Quality of Life questionnaire (AQoL-8D) [36, 37]	The AQoL-8D consists of 35 items across 8 dimensions, with higher scores within each dimension corresponding to poorer quality of life. Utility weighted AQoL-8D scores will be used to estimate quality-adjusted life years (QALYs) for the cost-effectiveness analysis [38].
Self-management behaviours	Questionnaire specifically designed for this study	Examples of self-management behaviours will include, but are not limited to, the use of heat packs or hot showers for LBP, massage (not delivered by a professional), brace or support strapping/tape, topical creams/gels, physical activity and exercise, relaxation, meditation, mindfulness techniques, and walking aids specifically used to manage LBP.
Medication use	Questionnaire specifically designed for this study	Data on the use of medications for managing LBP, including type (i.e., paracetamol, non-steroidal anti-inflammatory drugs, opioids), dosage, and whether the medication was prescribed by a medical or health professional, will be collected on a fortnightly basis.
Sleep quality	Pittsburgh Sleep Quality Index (PSQI) [39]	The PSQI is an 18-item self-reported questionnaire assessing sleep disturbances in the last month. The total score is composed of a sum of scores in 7 different domains and ranges from 0 to 21, with higher scores indicating poorer sleep quality.
Attitudes regarding use of pain medications	Short-form Pain Medication Attitudes Questionnaire (PMAQ-14) [40]	The PMAQ-14 consists of 14 items across 7 areas of concern for users of pain medications (addiction, need, scrutiny, side effect, tolerance, mistrust of doctors, withdrawal). Each item is scored on a 6-point scale with 0 representing never true and 5 representing always true.
Beliefs about back pain	Back Beliefs Questionnaire (BBQ) [41]	The BBQ consists of 14 items and total scores range from 9 to 45, with lower scores indicating more negative beliefs about back pain.

### Recruitment

#### Participant identification

Physiotherapists from the outpatient musculoskeletal physiotherapy departments of participating hospital sites will identify (all) patients who are within 1–2 weeks of their anticipated discharge from physiotherapy treatment for chronic LBP. The physiotherapist will introduce the study to all candidate patients. For patients indicating interest in participation, their contact details will be provided to the research team. The research team will phone the potential participant to provide them with further information about the study, including the study documents. Those who confirm their interest in the trial will be invited to take part in the consent procedures with a research team member, which will ideally occur within 1–2 weeks prior to discharge from treatment. De-identified demographic data (age, sex) and LBP pain scores at discharge from treatment (assessed with the NRS) will be collected from all candidate patients, irrespective of their interest or provision of consent. Data will be used to assess selection bias and target population reach.

#### Consent and screening

Depending on participant preference, consent procedures will occur face-to-face at the hospital site or remotely via an online consent form. For participants choosing to consent remotely, a research team member will provide support via phone call or video-conferencing software. During consent procedures, a research team member will discuss the study Participant Information Sheet and Consent Form with the participant (see Additional file 6). Potential participants who agree to take part in the study will be asked to sign a paper or online version of the study consent form. After signing the study consent form, the participant will be enrolled into the study and immediately assigned a unique participant code, which will be used on all study documents to protect their privacy. In addition, the research team will request written consent from participants to access their Medicare Benefits Schedule and Pharmaceutical Benefits Scheme claims data.

After consent procedures are completed, participants will be formally screened for eligibility and medical safety. For participants who meet the study eligibility criteria and are considered medically safe to participate in the trial, a research team member will phone the participant to invite them to take part in the baseline assessment. In accordance with standard Get Healthy Service® registration requirements [22], participants who are identified as requiring additional medical clearance will be referred to seek written recommendations from their medical doctor prior to randomisation.

### Baseline assessment

Depending on participant preference, baseline assessment will occur face-to-face at the hospital site with a research assistant, or remotely. For participants completing baseline assessment remotely, a research team member will be available to provide support via phone call or video-conferencing software. Participants will be required to complete an online (electronic) baseline questionnaire to record demographic and anthropometric data (age, height, weight), and the study outcomes (see Additional file 3). In addition, participants will be given a tri-axial accelerometer (Axivity) to wear for 7 consecutive days on their right thigh. During the 7-day period, participants will be asked to document any physical activities or exercises completed in a paper-based logbook (see Additional file 7). After 7 days, participants will return the accelerometer and logbook to the research team via a pre-paid reply envelope. At baseline assessment, all participants will also receive a paper-based weekly diary (see Additional file 8) to document any adverse events which may occur during the intervention period (6 months) - see below. For participants who complete the baseline assessment at the hospitals, the online baseline questionnaire will be completed on-site and the research team member will directly attach the accelerometer onto the participant’s thigh. For participants who complete the baseline assessment remotely, they will receive a link to the online baseline questionnaire via SMS or email, and any relevant study documents and equipment will be posted to them. The participant will receive a detailed sheet with labelled images and instructions on how to self-attach the accelerometer device (see Additional file 7). As required, a research team member will be available to provide support for any baseline assessment procedures, via phone call or video-conferencing software.

### Randomisation

After baseline assessment has been completed and the participant has been discharged from treatment, participants will be randomised with 1:1 allocation ratio to either the support system or usual care group. Treatment allocation will be performed using a computer-generated random allocation schedule operated by a remote unblinded researcher to ensure concealment. As participants will be randomised after discharge from treatment, with minimal potential for treatment contamination, randomisation will be at participant level. Randomisation will be by random permuted blocks of 4 and 6. Participants will be notified of their allocation via phone call from an unblinded research assistant. Select members of the research team will be unblinded to treatment allocation (i.e., main trial co-ordinator, the designated investigator managing referrals to the Get Healthy Service®, unblinded statistician monitoring trial safety, health coaches and agents from the Get Healthy Service®). Otherwise, all other research team members will remain blinded to treatment allocation. Participants will not be blinded to group allocation.

### Usual care group

Participants in the usual care group will only receive the standard care delivered by their physiotherapist at discharge from outpatient treatment from participating hospitals. Usual care commonly involves the provision of advice, education, and a home-based exercise program, with no further intentional follow-up appointments arranged. Standard usual care at discharge may also involve referral to local community exercise providers, although the availability of these programs is highly variable across hospital sites and community regions. Participants in the usual care group will be able to continue seeking other forms of health care and treatments as desired.

### Intervention group (support system involving referral to the Get Healthy Service®)

Similar to the usual care group, participants in the intervention group will receive the standard care delivered by their physiotherapist at discharge from outpatient treatment and will be able to seek other forms of health care and treatments as desired. In addition, participants in the intervention group will be referred for enrolment into the Get Healthy Service® *Standard Coaching* module (health coaching program) with a physical activity goal.

#### Referral to the Get Healthy Service®

All participants who are randomised to the support system will be referred to the Get Healthy Service® via the same structured referral pathway. A designated investigator (clinician) at each hospital will manage the referral process for the site. The designated investigator will enter the contact information of participants randomised to the support system group into a digital referral channel on the Get Healthy Service® website, via an online form specifically designed for the study. Evidence of medical clearance will be provided to the Get Healthy Service® via a secure file transfer program, when required. Upon receipt of the referral, a staff member (intake specialist) from the Get Healthy Service® will phone trial participants to complete the registration call. During the registration phone call, the intake specialist will enrol the participant into the *Standard Coaching* module. After successful enrolment, an assigned health coach will phone the participant to commence the health coaching program.

#### Structure of the Get Healthy Service®

The Standard Coaching module will be delivered in accordance with its current features and specifications, but with some adaptation to align the content of the module with best practice for management of LBP. Participants will be offered up to 10 individually tailored phone-based health coaching sessions over a 6-month period. Sessions will be approximately 17 minutes in duration and will be led by health coaches with university-qualifications in allied health (i.e., dietetics and/or exercise physiology). All health coaches are required to complete training in health coaching, predominately acquired through HealthChange® Australia (2-day Core Training program) [42], as well as internal training by clinical specialists as required. The sessions will be delivered to participants by their personal health coach, selected to match their goals and personal preference (i.e., male or female coach). The frequency of sessions will be tapered over the 6-month period according to each participant’s preference and progress through the program. The initial health coaching session will focus on mutually establishing the participant’s physical activity goals, as well as other health-related goals (i.e., reducing weight, achieving a healthy diet, reducing alcohol consumption) that are meaningful to the participant.

#### Contents of the health coaching program

Overall, the health coaching sessions will focus on utilising principles of behaviour change and self-regulation to assist participants with:

1. Increasing physical activity: Health coaches will assist participants to develop a tailored physical activity plan suitable to their individual lifestyle preferences;
2. Decreasing sedentary behaviour: Participants will be encouraged to increase daily incidental physical activity and decrease sitting time;
3. Achieving their health-related goals: The health coach will provide ongoing support and motivation to help participants achieve their personal physical activity and health-related goals. Health coaches will continually review participant progress with achieving their goals and assist with adjusting goals if necessary or as desired by the participant.

The contents of the *Standard Coaching* module will be individually tailored to meet the needs for people with chronic LBP. The tailored content will be informed by evidence-based recommendations for managing chronic LBP (see Table 3).

**Table 3:** Tailored health coaching content for chronic LBP

**Tailored health coaching content for chronic LBP**	
Goal-setting:	
• Mutually establish a physical activity goal with the participant at commencement of the health coaching program. Where relevant, this will include ongoing adherence to the exercise program prescribed by their hospital physiotherapist prior to discharge from treatment.	
• Establish other health-related goals that are meaningful to the participant (i.e., reducing weight, achieving a healthy diet, reducing alcohol consumption).	
Promotion of exercise and physical activity:	
• Explore barriers to exercise and physical activity participation (e.g., time, access, financial resources, social comfort).	
• Promote participant-led problem-solving skills to encourage overcoming perceived and real barriers to exercise or physical activity participation.	
Support:	
• Empower patients to foster self-efficacy and take charge of their own health, including monitoring their own symptoms and capacity to adhere to goals.	
• Encourage involvement of family members, partners, or friends for social support with achieving goals.	
• Provide continual motivation, encouragement, and support for the use of positive self-management strategies (e.g., physical activity, exercise).	
Interpersonal skills:	
• Build report, trust, and commonality with the participant.	
• Directly involve the participant in the problem-solving and decision-making processes.	
• *Educate and advise participants that the presence of pain does not always equal to harm.*	
Education:	
• *Educate and advise participants that many findings on imaging are common and do not necessarily identify the exact cause of pain. Further, imaging should only be carried out when consideration of serious pathology is clinically indicated.*	
• Identify and address unhelpful beliefs about their condition or progress.	
• *Educate and advise participants on the benefits of exercise and the consequences of inactivity such as prolonged bed rest (*i.e.*, muscle weakness).*	
• *Assist participants in navigating decision-making processes surrounding whether additional care from hospital, medical, or health services for LBP is necessary.*	
Pacing and activity modification:	
• *Encourage participants to maintain engagement in usual activities (*e.g.*, occupational, leisure).*	
• *Promote activity modification when required (*i.e.*, regress the difficulty of an exercise or activity, perform alternative exercises or tasks that do not elicit painful symptoms, minimise sustained repetitive postures and activities, minimise excessive loads when sitting, bending, or twisting).*	
• Educate and advise participants on incidental opportunities to increase physical activity levels when exercise may not be feasible (e.g., use public transportation, walk to the shops, stand at work, spend less time sitting at home).	
• *Encourage activity pacing when required, according to the participant’s physical capacity and goals.*	
Identifying and addressing psychological factors:	
• *Screen and address common psychological factors in chronic LBP populations (*e.g.*, fear avoidance, catastrophising, familial and social stress, work pressures, financial pressures).*	
• De-escalate potential perceived threats.	
• Ask simple and unambiguous questions.	
• *Avoid using catastrophising terms when discussing pain (*e.g.*, bulging disc, crumbling discs, degenerated discs).*	
• Use positive, supportive, and empathetic language.	
Reframing:	
• Focus problem-solving on the participant’s functional ability (i.e., improved ability to complete certain tasks or activities), instead of drawing attention to their pain.	
• Focus on activities that the participant can perform and what they are willing to try.	
• *Encourage participants to continue safe participation in exercise, even in the presence of acute symptoms (*i.e.*, flare-ups of LBP).*	
• Focus on activities that the participant has been able to perform successfully and provide ongoing encouragement for future success.	

Items in italics indicate content which has been tailored specifically for chronic LBP

After completing the *Standard Coaching* module, participants will be given the option to discontinue the program (i.e., graduation), re-enrol for further health coaching sessions, or opt into a free SMS maintenance support program *(Get Healthy Stay Healthy)* for an additional 6 months. The option selected by the participant will be recorded. For participants who opt into the *Get Healthy Stay Healthy* maintenance support program, they will receive automated, standardised (pre-scripted) motivational SMS reminders tailored towards 3 distinct goal categories: (1) physical activity, (2) diet, (3) weight maintenance. Participants will be asked to select one-to-two goal categories of interest and indicate their preferences for the SMS reminders (i.e., frequency of receiving reminders, number of reminders received per goal category). Their personal health coach will establish specific behaviour goals (e.g., walk for 30 min daily), personal barriers (e.g., distracted and often miss walking time), and enablers (e.g., set phone alarm for 6 pm daily), which will be embedded into the SMS messages. At 3 months into the *Get Healthy Stay Healthy* program, the health coach will phone the participant to monitor their progress and adjust the goal/s or reminder preferences as needed. At 6 months, the health coach will phone the participant to confirm completion from the SMS program (i.e., graduation) and encourage ongoing self-maintenance of positive health behaviours.

### Training

A series of training workshops will be implemented. Physiotherapists from the outpatient musculoskeletal physiotherapy departments of participating hospital sites will receive training from the research team to upskill on the process of identification of interested patients to the research team. The research team will undergo training to upskill on recruitment procedures (i.e., consent, eligibility screening). The designated investigator responsible for coordinating referral of participants to the Get Healthy Service® at each hospital site will receive training for managing the process (i.e., use of the digital referral form, provision of evidence of medical clearance). Health coaches delivering the study intervention will undergo a training workshop to familiarise themselves with the health coaching content which has been tailored to match the needs of chronic LBP (see Table 3). Health coaches will also receive training regarding indicators and procedures for clinical escalation (see Additional file 9).

### Assessment of intervention fidelity and engagement

#### Fidelity of the referral process

We will assess fidelity of the referral process by recording the total number of participants who are randomised to the support system and successfully enrolled into the Get Healthy Service® (i.e., completion of registration call) and expressing this as a percentage of the total number of participants are randomised to the support system group.

#### Fidelity of the health coaching program

The Get Healthy Service® conducts internal health coaching call audits for 1% of all calls per month carried out by the service. The audits are based on the following key criteria: security and privacy, clinical best practice, health coaching (i.e., effective utilisation of health coaching skills and techniques), call etiquette, documentation. Further, in the initial period of study implementation, the Get Healthy Service® will perform targeted auditing of health coaching calls delivered for participants specifically referred from the trial. The purpose will be to ensure that appropriate clinical best practice and high quality referral processing are achieved.

The research team will assess fidelity of the health coaching program by evaluating the following: (i) number of participants randomised to the support system who successfully establish a physical activity goal at commencement of the health coaching program; (ii) total number of health coaching sessions received per participant; (iii) number of participants who complete the Get Healthy Service® *Standard Coaching* module, where completion will be defined as receiving the 10 allocated health coaching sessions or achievement of participant goals. Data will be collected by the Get Healthy Service® and provided to the research team.

#### Participant engagement

We will assess participant engagement with the study intervention by recording the total number of participants who successfully complete the *Standard Coaching* module and expressing this as a proportion of the total number of participants who are successfully enrolled into the *Standard Coaching* module (tailored for chronic LBP).

### Monitoring adverse events

The research team has designed this study to minimise or prevent potential risks. Expected adverse events include: i) flare-ups of LBP, ii) muscle soreness, swelling, or muscle cramps related to commencement of unaccustomed exercise; iii) unexpected trip/fall. During the intervention period, participants will complete a paper-based weekly diary to capture any adverse events which may occur during the 6-month intervention period (see Additional file 8). Participants will be required to document any adverse events by answering the question: ‘Did you experience any of the following this week?’. Possible responses will include increased back pain, pain elsewhere, muscle soreness, swelling, muscle cramp, trip/fall, emotional distress, serious event, and other symptoms. If relevant, participants will be asked whether the event persisted more than 24 h (yes or no) and whether medical attention was sought (yes or no). If the participant perceives that the adverse event is directly related to participation in the study or they have ongoing unresolved concerns about the event, the diary contains written instructions directing them to contact the research team as soon as possible. The research team will monitor the adverse event until resolution. If participants are harmed from taking part in the study, there will be no special compensation arrangements.

### Follow-up data collection

At 6 and 12-months from baseline assessment, participants will complete a follow-up online (electronic) questionnaire to record the study outcomes (see Additional file 4). At each timepoint, participants will receive the link to complete the respective online questionnaire via SMS or email, depending on their preference. At the 6-month follow-up, the participant will also repeat the procedure of wearing the accelerometer device and completing the physical activity logbook for 7 consecutive days. Participants will receive the accelerometer, an instruction sheet for self-mounting the device, the logbook, and a pre-paid reply envelope via post.

All participants will complete a brief questionnaire every fortnight during the 12-month assessment period (see Additional file 5). Participants will receive a link to complete the online fortnightly questionnaire via SMS or email. The questionnaire will collect self-reported data related the primary outcome (i.e., use of hospital, medical, and health services for LBP), and select secondary outcomes (i.e., pain intensity, self-management behaviours). Participants will be asked whether they experienced LBP (yes or no) and whether they sought any care or treatment for LBP (yes or no), in the past fortnight. Where relevant, follow-up questions will be asked regarding the mean pain intensity and number of days experiencing LBP, the type of care or treatment sought, and any costs or travel time associated with managing their LBP.

In addition, an unblinded member of the research team will briefly contact all participants at 3, 6 and 9 months to identify any concerns of the participant regarding participation in the study, such as potential barriers towards ongoing engagement with responding to fortnightly and follow-up questionnaires. The research team will use this information to develop strategies to promote ongoing study engagement (e.g., explain to participants the importance of obtaining a complete data set for the study). To maintain engagement with participants in the usual care group, participants in the usual care group will be offered the opportunity to enrol into any of Get Healthy Service’s® programs at the completion of their 12-month follow-up assessment.

### Monitoring contamination

The Get Healthy Service® is well-established and free to use for state residents. It is possible that participants in the usual care group may self-refer to the Get Healthy Service® and enrol in any of the health coaching programs offered. To minimise the risk of contamination, participants will be informed prior to study enrolment that those randomised to the usual care group will be asked to not participate in the health coaching intervention. Participants will also be informed that at the end of their participation in the trial (i.e., after completing their 12-month follow-up assessment), they will be offered the opportunity to enrol into the Get Healthy Service® and any of its programs. During the trial, participants will still be allowed to seek other forms of care or continue with the standard usual care provided to them by their physiotherapist at discharge from treatment.

If a participant in the usual care group self-enrols into any of the Get Healthy Service's® programs during the study period, we will not ask them to discontinue the program and will continue collecting data from them unless they choose to withdraw from the study entirely. At the conclusion of the study (i.e., after completion of the 12-month follow-up), a research team member will phone all participants in the usual care group to confirm whether they self-referred to any of the Get Healthy Service® programs during the intervention period. During this phone call, participants who express subsequent interest in the Get Healthy Service® will be offered enrolment into the service.

### Embedded qualitative study

We will conduct an embedded qualitative study of a sample of key stakeholders (including clinicians, trial participants, and health coaches and agents from the Get Healthy Service®) involved in the study. The purpose will be to identify factors related to the intervention, context, and individual, to inform the development of an implementation plan for scalability of the approach across New South Wales local health districts.

We will conduct a series of in-depth interviews and one or more focus groups to identify factors and processes that contributed to the program outcomes. The interviews and focus group(s) will be informed by the Consolidated Framework for Implementation Research (CFIR) [43], and will focus on exploring the following CFIR constructs: (1) intervention characteristics (e.g., relative advantage, adaptability, cost); (2) outer setting (e.g., patient needs and resources, external policies and incentives); (3) inner setting (e.g., implementation climate, relative priority, available resources); (4) characteristics of individuals (e.g., knowledge and beliefs about the intervention, individual identification with organisation); (5) processes (e.g., executing, engaging, reflecting and evaluating).

Participants will be purposively selected to ensure a range of demographics, health services, and experiences are captured, and interviews will continue until theme saturation is reached. We expect to conduct in-depth interviews with approximately 30 clinicians and trial participants, and a focus group involving health coaches and agents from the Get Healthy Service®. A mixture of inductive and deductive (drawing on the CFIR) interview analysis will be undertaken. Key themes will be used to guide development of recommendations for scalability of the support system.

### Data integrity and monitoring

A Data Monitoring Committee (DMC) will be convened to overview data collection and integrity. The DMC will approve the statistical analysis plan and research protocol. Interim analyses of baseline data may be undertaken, under the guidance and approval from the DMC. The integrity of trial data will be monitored by regularly scrutinising data sheets for omissions and errors. Data inconsistencies will be explored and resolved. The lead investigator will be responsible for overseeing trial safety and ensuring that the best interests of participants are observed at all times. The lead investigator will be blinded to allocation, unless unblinding is deemed essential to ensure participant safety. Adverse events will be reported to the reviewing Human Research Ethics Committee and study Sponsor in accordance with approved requirements. All data collected will be restricted to the lead investigator and select members of the research team.

### Protocol amendments

Any modifications to the protocol will be submitted to the reviewing Human Research Ethics Committee and acknowledged by the trial sponsor before implementation. Amendments will be communicated to the relevant trial registries and included in publications of trial results.

### Sample size calculation

The sample size is that required to detect a clinically meaningful between-group difference in the primary outcome, i.e., the total number of encounters with hospital, medical, and health services for LBP [44]. A total of 374 participants (*n* = 187 per group) will be recruited. The study will have 90% power to detect as significant, at the 5% level, a 30% difference in the rate of using hospital, medical, and health services for LBP between groups (i.e., an Incidence Rate Ratio of 0.70 using negative binomial regression analysis) over the 12-month study period. The 30% difference in the rate of using hospital, medical, and health services for LBP is based on our research of patients’ perceptions of a clinical worthwhile effect of interventions for LBP [45]. Estimates were based on a base rate exposure (β0) of 0.2 [total of 2 care-seeking events in the usual care group per fortnight (data from pilot)], assuming a correlation of 0.3 (R^2^ = 0.09) between covariates and predictor (treatment), using negative binomial regression model (G*Power® software) [46]. Estimates allow for a loss to follow-up of 10%.

### Statistical analysis

The total number of encounters with hospital, medical, and health services for LBP per person, over 12 months from baseline assessment, will be analysed using negative binomial regression to estimate the between-group difference in the rate of using hospital, medical, and health services for LBP at 12-month follow-up. Negative binomial regression takes into account individual follow-up time, frequency of using hospital, medical, and health services for LBP, non-normal distribution over time, and non-independence of repeated measures [44]. The effect of baseline pain and disability levels, number of previous treatments, symptom length, co-morbidities, and age will be accounted for in the model. The effect of group allocation on continuous outcomes (e.g., physical function, physical activity) will be assessed using linear regression models. All analyses will be performed by intention to treat.

### Health system resource use and costs

Each episode of using hospital, medical, and health services for LBP for all randomised participants will be identified through study records and valued using Australian Refined Diagnosis Related Groups (AR-DRG) cost weights and Net Efficient Pricing for in-patient admissions; and MBS items for outpatient care (e.g., health care visits, tests, procedures). Prescribed medicines will be identified and valued from PBS claims. In addition, we will collect study-related costs for the Get Healthy Service® health coaching intervention and its implementation (e.g., staff salary, consumables) and delivery of usual care. Total costs and mean (standard deviation) per patient costs by allocation at 6 and 12 months will be tabulated and compared. The difference in health care use, and costs between groups will be reported with 95% confidence intervals.

### Economic evaluation

A within-trial cost-effectiveness analysis from an Australian health system perspective will be undertaken. The measure of effectiveness will be the quality-adjusted life year (QALY) based on utility weights from the AQoL-8D questionnaire and participant survival at 12 months. Quality of life data will be assessed for missingness, and imputation methods will be employed if appropriate. Mean per patient and total utilities and QALYs will be tabulated by allocation, with precision estimates for differences between groups. An incremental cost effectiveness ratio (ICER) will be calculated from the difference in costs (health care use) and QALYs gained. A confidence limit around the ICER will be calculated using a non-parametric bootstrapping approach. The probability of the support system being cost-effective will be assessed at different willingness to pay levels and plotted on a cost-effectiveness acceptability curve. Scenario analyses will be undertaken to explore cost-effectiveness for specific populations (e.g., by sex). The economic evaluation will follow best practice recommendations with further details in the health economics analysis plan (HEAP) [47].

### Trial status

Trial recruitment will commence in July 2021. The current protocol is version 6, dated 26 May 2021.

### Confidentiality

The confidentiality of participants and privacy of data will be protected during all publications, presentations, and dissemination activities. Data will be presented as summary statistics such that individual participants will not be identifiable in the research reports or presentations.

### Dissemination policy

The research team will provide participants with a summary of the study findings in lay language. Study results will be submitted for publication in reports and peer-reviewed journals. Study results will also be presented in a variety of conferences and forums, targeting both researchers and the general community. All investigators will be considered for authorship on future publications in accordance with their contributions.

## Discussion

Current models of care for LBP in the public health care systems of most countries, including Australia, lacks the capacity to support people with LBP after discharge from treatment. Whilst most patients achieve continual improvements after treatment cessation [7], approximately one in five experience worsening symptoms after discharge and seek further care again within 12 months [10, 11]. The economic and resource burden imposed on health care systems by this cyclical pattern is substantial, highlighting the persistence of an important gap in the chronic LBP clinical care pathway (i.e., abrupt therapeutic void and lack of support after treatment discharge).

From consultations with senior musculoskeletal clinicians and consumer groups representing patients with LBP, we identified that lack of a structured support system after completing hospital-based outpatient physiotherapy treatment for chronic LBP was considered a strong driving factor for patients seeking further treatment. This is consistent with findings from a systematic review examining perceived health information needs related to LBP: patients with LBP strongly express the desire to receive clear information about the ongoing availability of medical and allied health services, non-medical support from social networks and support groups, or work-specific support services [48]. Similarly, a qualitative study of patient perceptions of self-managing chronic LBP following discharge from physiotherapy care also identified a strongly perceived need for self-management support following discharge from treatment (i.e., direct access and/or review appointments, telephone calls) [49]. Together, these studies highlight that patients with LBP consistently desire the availability or awareness of support services after cessation of treatment; although, targeted patient education should be provided to contextualise the appropriate use of further medical and allied health services.

Currently in Australian public hospitals, local health district-driven approaches to support people with LBP after completion of hospital outpatient physiotherapy care are inconsistent, poorly coordinated, and generally lacking. Some hospitals may offer short-term general exercise classes or refer patients to local community exercise programs. However, the availability of such programs is highly variable across different hospitals and communities, and it is atypical for patients to receive further intentional follow-up appointments or support from their physiotherapist. Systematic reviews of international clinical guidelines and care pathways suggest that this pattern is similar globally. Guidelines also lack consistency in recommendations for provision of ongoing patient support [50, 51]. The existing model of care for chronic LBP globally appears insufficient to meet patient needs after treatment cessation.

There is an increasing global need to develop solutions for LBP that move beyond medicalised approaches for LBP [52]. Improved solutions for chronic LBP should focus on incorporating conservative strategies that link education about LBP with sustainable positive lifestyle changes and pain-coping behaviours [48, 52]. In particular, the proposed solution should also take into consideration patient preferences, including the provision of consistent, high-quality, tailored education regarding self-management strategies and the availability of support services for LBP [48]. Further, there is increasing emphasis that care for chronic pain conditions, such as LBP, should be grounded in the community [53]. Taken together, it seems that a supported self-management approach which incorporates community-based services may be a promising choice. The implementation of a simple, low-cost but well-structured post-discharge support service into the clinical care pathway for LBP could fill this gap.

Health coaching is an innovative and viable solution with strong potential to support LBP patients after discharge from treatment. Evidence from our pilot study supports that telephone-based health coaching can result in clinically important improvements in physical activity in patients with chronic LBP [18] and reduce the rate of care-seeking for the condition by 38% [95% confidence interval 0.32 to 1.18], compared with usual post-discharge management [11]. The Get Healthy Service® currently offers an established, well-structured, telephone-based health coaching program which focuses on supporting participants to develop self-efficacy in increasing physical activity levels, reducing sedentary behaviour, and achieving sustainable patient-centred goals. The Get Healthy Service® is fully funded by the New South Wales Ministry of Health and is free to use for state residents of three states [22]. In this study, we will test whether the introduction of a support system at discharge from hospital outpatient physiotherapy treatment for chronic LBP (involving a coordinated referral pathway linking hospital-based outpatient physiotherapy services to the Get Healthy Service®), reduces the number of re-presentations to medical, hospital, or health care services for LBP, as evidence of better support for maintenance of clinical improvements.

This manuscript presents the rationale and design of a RCT testing a novel support system which involves a structured referral pathway linking hospital-based outpatient physiotherapy services for chronic LBP to a health coaching program delivered by the Get Healthy Service®. The contents of the health coaching program will be tailored to meet the needs of people with chronic LBP. The support system will be compared with the usual care provided at discharge from outpatient physiotherapy care at each participating hospital site. Findings will evaluate the effect of the support system on the future use of hospital, medical, and health services for LBP, in people recently discharged from hospital outpatient physiotherapy treatment for chronic LBP and test the effectiveness and cost-effectiveness of the support system for improving LBP symptom-related outcomes and behaviours (pain, disability, physical activity levels, quality of life). We also describe an embedded qualitative study designed to identify factors related to the intervention, context, individual, and implementation process which are likely to contribute to intervention success or otherwise. If positive, findings will inform the development of an implementation plan for scaling-up this approach, which could be disseminated across other health districts. Further, study findings could be disseminated across the general community to increase consumer awareness of the availability of physical activity-focused health coaching programs, which can be readily integrated into the discharge care pathway for patients receiving treatment for LBP.

## Conclusion

Community-driven solutions that support people with chronic LBP to better self-manage their condition and potentially reduce their use of further hospital, medical, and health services, after discharge from treatment, are urgently needed. The proposed study will test a novel support system which involves a structured referral pathway that directly links hospital-based outpatient physiotherapy services to the Get Healthy Service®. If positive, study findings will help to inform health care policy and clinical practice for chronic LBP in Australia.

## Supplementary Information

**Additional file 1.**

**Additional file 2.**

**Additional file 3.**

**Additional file 4.**

**Additional file 5.**

**Additional file 6.**

**Additional file 7.**

**Additional file 8.**

**Additional file 9.**

## Data Availability

Not applicable.

## Abbreviations

AQoL: Assessment of Quality of Life
AR-DRG: Australian Refined Diagnosis Related Groups
BBQ: Back Beliefs Questionnaire
CFIR: Consolidated Framework for Implementation Research
CONSORT: CONsolidated Standards Of Reporting Trials
DMC: Data Monitoring Committee
GPAQ: Global Physical Activity Questionnaire
HEAP: health economics analysis plan
ICER: incremental cost effectiveness ratio
LBP: low back pain
NRS: Numerical Rating Scale
PMAQ: Pain Medication Attitudes Questionnaire
PSFS: Patient Specific Functional Scale
PSQI: Pittsburgh Sleep Quality Index
QALY: quality-adjusted life year
RMDQ: Roland–Morris Disability Questionnaire
SPIRIT: Standard Protocol Items: Recommendations for Intervention Trials
TIDieR: Template for Intervention Description and Replication
