# Supplementary material for: Effectiveness of a coordinated support system linking public hospitals to a health coaching service compared with usual care at discharge for patients with chronic low back pain: protocol for a randomised controlled trial
Source: BMC Musculoskelet Disord. 2021 Jul 9;22:611. doi: 10.1186/s12891-021-04479-z (PMC8272287; doi:10.1186/s12891-021-04479-z)
Supplement: Supplementary file 4 — Additional file 4. [file 12891_2021_4479_MOESM4_ESM.docx]

**6-MONTH AND 12-MONTH FOLLOW-UP QUESTIONNAIRE**

We expect that this questionnaire will take approximately 20-30 minutes to complete. There is an option to save your responses and return to the questionnaire later if you are unable to complete immediately.

**SECTION 1: IDENTIFICATION**

| **1.** Date: ____ / ______/ _______  **2.** Participant study ID: ________________________________ |
| --- |
| **3**. What is your sex? ❑ Male ❑ Female |

**SECTION 2: LOW BACK PAIN HISTORY**

**1**.In the last 6 months, have you had surgery for your lower back pain?

❑ No 🡪 *Skips to LOW BACK PAIN SYMPTOMS*

❑ Yes

**2.** Which type of surgery? ❑ Microdiscectomy ❑ Discectomy ❑ Laminectomy/decompression

❑ Fusion ❑ Unsure ❑ Other, please specify__­­­___________

**SECTION 3: LOW BACK PAIN SYMPTOMS**

We would like to know about the intensity of your low back pain symptoms. Please answer the following questions.

**Low Back Pain Intensity (Current)**

**1**. Please rate the intensity of your current low back pain today, where 0 = no pain and 10 = worst possible pain.

No pain

Moderate

Pain

Worst possible Pain

0

1

2

3

4

5

6

7

8

9

10

**Low Back Pain Intensity (In the Past Fortnight)**

**2.** Please rate the intensity of your average low back pain over the past fortnight, where 0 = no pain and 10 = worst possible pain.

No pain

Moderate

Pain

Worst possible Pain

0

1

2

3

4

5

6

7

8

9

10

**Low Back Pain Frequency (In the Past Fortnight)**

**3.** Over the last 2 weeks (fortnight), how many days did you experience low back pain?_____________________

**Low Back Pain Disability (Roland Morris Disability Questionnaire)**

We would like to know about any disability caused by your low back pain. Please answer the following questions.

**4.** The purpose of the following questions is the understand how much your low back pain interferes with your daily activities. Please select yes or no to the following questions.

|  | 1. I stay at home most of the day because of the pain in my back | ❑ No ❑ Yes |
| --- | --- | --- |
|  | 1. I change position frequently to try and get my back comfortable. | ❑ No ❑ Yes |
|  | 1. I walk more slowly than usual because of the pain in my back. | ❑ No ❑ Yes |
|  | 1. Because of the pain in my back, I am not doing any of the jobs that I usually do around the house. | ❑ No ❑ Yes |
|  | 1. Because of the pain in my back, I use a handrail to climb stairs. | ❑ No ❑ Yes |
|  | 1. Because of the pain in my back, I lie down to rest more often than usual. | ❑ No ❑ Yes |
|  | 1. Because of the pain in my back, I have to hold on to something to get out of a lounge chair. | ❑ No ❑ Yes |
|  | 1. Because of the pain in my back, I ask other people to do things for me. | ❑ No ❑ Yes |
|  | 1. I get dressed more slowly than usual because of the pain in my back. | ❑ No ❑ Yes |
|  | 1. I only stand up for short periods of time because of the pain in my back. | ❑ No ❑ Yes |
|  | 1. Because of the pain in my back, I try not to bend or kneel down. | ❑ No ❑ Yes |
|  | 1. I find it difficult to get out of a dining chair because of the pain in my back. | ❑ No ❑ Yes |
|  | 1. My back is painful most of the time. | ❑ No ❑ Yes |
|  | 1. I find it difficult to turn over in bed because of the pain in my back. | ❑ No ❑ Yes |
|  | 1. I do not feel like eating much because of the pain in my back. | ❑ No ❑ Yes |
|  | 1. I have trouble putting on my socks (or stockings) because of the pain in my back. | ❑ No ❑ Yes |
|  | 1. I only walk short distances because of the pain in my back. | ❑ No ❑ Yes |
|  | 1. I sleep less than usual because of the pain in my back. | ❑ No ❑ Yes |
|  | 1. Because of the pain in my back, I get dressed with help from someone else. | ❑ No ❑ Yes |
|  | 1. I sit down for most of the day because of the pain in my back. | ❑ No ❑ Yes |
|  | 1. I avoid heavy jobs in the house because of the pain in my back. | ❑ No ❑ Yes |
|  | 1. Because of the pain in my back, I am more irritable and bad tempered with people than usual. | ❑ No ❑ Yes |
|  | 1. Because of the pain in my back, I climb stairs more slowly than usual. | ❑ No ❑ Yes |
|  | 1. I stay in bed most of the time because of the pain in my back. | ❑ No ❑ Yes |

**SECTION 4: PHYSICAL ACTIVITY LEVELS**

**Global Physical Activity Questionnaire**

We would like to know about the time you spend doing different types of physical activity in a typical week. Please answer these questions even if you do not consider yourself to be a physically active person.

Think first about the time you spend doing work. Think of ‘work’ as the things that you have to do such as paid or unpaid work, study/training, household chores, seeking employment. In answering the following questions:

- 'Vigorous-intensity activities' are activities that require hard physical effort and cause large increases in breathing or heart rate
- 'Moderate-intensity activities' are activities that require moderate physical effort and cause small increases in breathing or heart rate.

**Work**

| **1.** Does your work involve vigorous-intensity activity that causes large increases in breathing or heart rate like [carrying or lifting heavy loads, digging or construction work] for at least 10 minutes continuously?  ❑ Yes ❑ No 🡪 *Skips to 4* |
| --- |
| **2.** In a typical week, on how many days do you do vigorous-intensity activities as part of your work?  Number of days _____________________ |
| **3.** How much time do you spend doing vigorous-intensity activities at work on a typical day?  Hours: minutes __________:___________ |
| **4.** Does your work involve moderate-intensity activity, that causes small increases in breathing or heart rate such as brisk walking [or carrying light loads] for at least 10 minutes continuously?  ❑ Yes ❑ No 🡪 *Skips to 7* |
| **5.** In a typical week, on how many days do you do moderate intensity activities as part of your work?  Number of days _____________________ |
| **6.** How much time do you spend doing moderate-intensity activities at work on a typical day?  Hours: minutes __________:___________ |

**Travel To and From Places**

The next questions exclude the physical activities at work that you have already mentioned. Now we would like to ask you about the usual way you travel to and from places (e.g. to work, for shopping, to market, to place of worship).

| **7.** Do you walk or use a bicycle (pedal cycle) for at least 10 minutes continuously to get to and from places?  ❑ Yes ❑ No 🡪 *Skips to 10* |
| --- |
| **8.** In a typical week, on how many days do you walk or bicycle for at least 10 minutes continuously to get to and from places?  Number of days _____________________ |
| **9.** How much time do you spend walking or bicycling for travel on a typical day?  Hours:minutes __________:___________ |

**Recreational Activities**

The next questions exclude the work and transport activities that you have already mentioned. Now we would like to ask you about sports, fitness and recreational activities (leisure).

| **10.** Do you do any vigorous-intensity sports, fitness or recreational (leisure) activities that cause large increases in breathing or heart rate like [running or football] for at least 10 minutes continuously?  ❑ Yes ❑ No 🡪 *Skips to 13* |
| --- |
| **11.** In a typical week, on how many days do you do vigorous-intensity sports, fitness or recreational (leisure) activities?  Number of days _____________________ |
| **12.** How much time do you spend doing vigorous-intensity sports, fitness or recreational activities on a typical day?  Hours: minutes __________:___________ |
| **13.** Do you do any moderate-intensity sports, fitness or recreational (leisure) activities that cause a small increase in breathing or heart rate such as brisk walking, [cycling, swimming, volleyball] for at least 10 minutes continuously?  ❑ Yes ❑ No 🡪 *Skips to 16* |
| **14.** In a typical week, on how many days do you do moderate intensity sports, fitness or recreational (leisure) activities?  Number of days _____________________ |
| **15.** How much time do you spend doing moderate-intensity sports, fitness or recreational (leisure) activities on a typical day?  Hours: minutes__________:___________ |

**Sedentary behaviour**

The following question is about sitting or reclining at work, at home, getting to and from places, or with friends including time spent sitting at a desk, sitting with friends, traveling in car, bus, train, reading, playing cards or watching television, but do not include time spent sleeping.

| **16.** How much time do you usually spend sitting or reclining on a typical day?  Hours: minutes _____________________ |
| --- |

**SECTION 5: FUNCTION**

**Patient Specific Functional Scale**

The purpose of this questionnaire is to ask you to identify up to three important activities that you are unable to do or are having difficulty with as a result of your low back pain.

Please answer the following questions by selecting one number from the following scoring scheme (out of 10):

Unable to perform activity

Able to perform activity at the same level as before injury or problem

0

1

2

3

4

5

6

7

8

9

10

|_

When we assessed you at baseline, you indicated that you had difficulty with the following activities.

Today, do you still have difficulty with:

| Activity | 6-month follow-up | 12-month follow-up |
| --- | --- | --- |
| **1a.** | ***1b.*** *Score:*    / 10 | ***1c.*** *Score:*    / 10 |
| **2a.** | ***2b.*** *Score:*    / 10 | ***2c.*** *Score:*    / 10 |
| **3a.** | ***3b.*** *Score:*    / 10 | ***3c.*** *Score:*    / 10 |

**SECTION 6: QUALITY OF LIFE**

We would like to know about your quality of life. Please answer the following questions.

Assessment of Quality of Life 8-D Questionnaire (AQOL-8D)

Tick the box that best describes your situation as it has been over the past week:

| **Q1** | How much energy do you have to do the things you want to do?  I am:  ❑ always full of energy  ❑ usually full of energy  ❑ occasionally energetic  ❑ usually tired and lacking energy  ❑ always tired and lacking energy. |
| --- | --- |
| **Q2** | How often do you feel socially excluded or left out?   \| ❑ Never \| ❑ Rarely \| ❑ Sometimes \| ❑ Often \|  \| \| --- \| --- \| --- \| --- \| --- \| |
| **Q3** | How easy or difficult is it for you to get around by yourself outside your place of residence (e.g. to go shopping, visiting)?  ❑ Getting around is enjoyable and easy  ❑ I have no difficulty getting around outside my place of residence  ❑ A little difficulty  ❑ Moderate difficulty  ❑ A lot of difficulty  ❑ I cannot get around unless somebody is there to help me |
| **Q4** | Does your health affect your role in your community (eg. Residential, sporting, church or cultural activities)?  ❑ My role in the community is unaffected by my health  ❑ There are some parts of my community role I cannot carry out  ❑ There are many parts of my community role I cannot carry out  ❑ I cannot carry out any part of my community role |
| **Q5** | How often do you feel sad?   \| ❑ Never \| ❑ Rarely \| ❑ Some of the time \| ❑ Usually \| ❑ Nearly all the time \| \| --- \| --- \| --- \| --- \| --- \| |
| **Q6** | How often do you experience serious pain?  I experience it:  ❑ Very rarely  ❑ Less than once a week  ❑ Once or twice a week  ❑ Three to four times a week  ❑ Most of the time |
| **Q7** | How much confidence do you have in yourself?  ❑ Complete confidence  ❑ A lot  ❑ A moderate amount  ❑ A little  ❑ None at all |
| **Q8** | Do you normally feel calm and tranquil or agitated? I am:  ❑ always calm and tranquil  ❑ usually calm and tranquil  ❑ sometimes calm and tranquil, sometimes agitated  ❑ usually agitated  ❑ always agitated |
| **Q9** | Does your health affect your relationship with your family?  ❑ My role in the family is unaffected by my health  ❑ There are some parts of my family role I cannot carry out  ❑ There are many parts of my family role I cannot carry out  ❑ I cannot carry out any part of my family role. |
| **Q10** | How satisfying are your close relationships (family and friends)?  ❑ Very satisfying  ❑ Satisfying  ❑ Neither satisfying nor dissatisfying  ❑ Dissatisfying  ❑ Unpleasant  ❑ Very unpleasant |
| **Q11** | How well do you communicate with others (talking, signing, texting, being understood by others and understanding them)?  ❑ I have no trouble being understood  ❑ I have some difficulty being understood by people who do not know me.  ❑ I am understood only by people who know me.  ❑ I cannot adequately communicate with others |
| **Q12** | How often do you have trouble sleeping?   \| ❑ Never \| ❑ Almost never \| ❑ Sometimes \| ❑ Often \| ❑ All the time \| \| --- \| --- \| --- \| --- \| --- \| |
| **Q13** | How often do you feel worthless?   \| ❑ Never \| ❑ Almost never \| ❑ Sometimes \| ❑ Usually \| ❑ Always \| \| --- \| --- \| --- \| --- \| --- \| |
| **Q14** | How often do you feel angry?   \| ❑ Never \| ❑ Almost never \| ❑ Sometimes \| ❑ Often \| ❑ All the time \| \| --- \| --- \| --- \| --- \| --- \| |
| **Q15** | How easy or difficulty is it for you to move around (using any aids or equipment you need e.g. a wheelchair, frame or stick)?  ❑ I am very mobile  ❑ I have no difficulty with mobility  ❑ I have some difficulty with mobility (for example, going uphill)  ❑ I have difficulty with mobility, I can go short distances only.  ❑ I have a lot of difficulty with mobility, I need someone to help me  ❑ I am bedridden |
| **Q16** | Do you ever feel like hurting yourself?   \| ❑ Never \| ❑ Rarely \| ❑ Sometimes \| ❑ Often \| ❑ All the time \| \| --- \| --- \| --- \| --- \| --- \| |
| **Q17** | How enthusiastic do you feel?   \| ❑ Extremely \| ❑ Very \| ❑ Somewhat \| ❑ Not much \| ❑ Not at all \| \| --- \| --- \| --- \| --- \| --- \| |
| **Q18** | How often did you feel worried in the last seven days?   \| ❑ Never \| ❑ Occasionally \| ❑ Sometimes \| ❑ Often \| ❑ All the time \| \| --- \| --- \| --- \| --- \| --- \| |
| **Q19** | How difficulty is it for you to wash, toilet, dress yourself, eat or care for your appearance?  ❑ These things are very easy for me to do  ❑ I have no real difficulty in doing these things  ❑ I find some of these things difficult, but I manage to do them on my own  ❑ Many of these things are difficult, and I need help to do them  ❑ I cannot do these things by myself at all |
| **Q20** | How often do you feel happy?   \| ❑ All the time \| ❑ Mostly \| ❑ Sometimes \| ❑ Almost never \| ❑ Never \| \| --- \| --- \| --- \| --- \| --- \| |
| **Q21** | How much do you feel you can cope with life’s problems?   \| ❑ Completely \| ❑ Mostly \| ❑ Partly \| ❑ Very little \| ❑ Not at all \| \| --- \| --- \| --- \| --- \| --- \| |
| **Q22** | How much pain or discomfort do you experience?  ❑ None at all  ❑ I have moderate pain  ❑ I suffer from severe pain  ❑ I suffer unbearable pain |
| **Q23** | How much do you enjoy your close relationships (family and friends)?   \| ❑ Immensely \| ❑ A lot \| ❑ A little \| ❑ Not much \| ❑ I hate it \| \| --- \| --- \| --- \| --- \| --- \| |
| **Q24** | How often does pain interfere with your usual activities?   \| ❑ Never \| ❑ Rarely \| ❑ Sometimes \| ❑ Often \| ❑ Always \| \| --- \| --- \| --- \| --- \| --- \| |
| **Q25** | How often do you feel pleasure?   \| ❑ Always \| ❑ Usually \| ❑ Sometimes \| ❑ Almost never \| ❑ Never \| \| --- \| --- \| --- \| --- \| --- \| |
| **Q26** | How much of a burden do you feel you are to other people?   \| ❑ Not at all \| ❑ A little \| ❑ A moderate amount \| ❑ A lot \| ❑ Totally \| \| --- \| --- \| --- \| --- \| --- \| |
| **Q27** | How content are you with your life?   \| ❑ Extremely \| ❑ Mainly \| ❑ Moderately \| ❑ Slightly \| ❑ Not at all \| \| --- \| --- \| --- \| --- \| --- \| |
| **Q28** | How well can you see (using your glasses or contact lenses if they are needed)?  ❑ I have excellent sight  ❑ I see normally  ❑ I have some difficulty seeing things sharply. (e.g. small print, objects in the distance, or watching television)  ❑ I have a lot of difficulty seeing sharply.  ❑ I only see general shapes.  ❑ I am completely blind |
| **Q29** | How often do you feel in control of your life?   \| ❑ Always \| ❑ Mostly \| ❑ Sometimes \| ❑ Only occasionally \| ❑ Never \| \| --- \| --- \| --- \| --- \| --- \| |
| **Q30** | How much help do you need with jobs around your place of residence (e.g. preparing food, cleaning  ❑ I can do all these tasks very easily without any help  ❑ I can do these tasks relatively easily without help  ❑ I can do these tasks only very slowly without help  ❑ I cannot do most of these tasks unless I have help  ❑ I can do none of these tasks by myself |
| **Q31** | How often do you feel socially isolated?   \| ❑ Never \| ❑ Rarely \| ❑ Sometimes \| ❑ Often \| ❑ Always \| \| --- \| --- \| --- \| --- \| --- \| |
| **Q32** | How well can you hear (using your hearing aid if needed)?  ❑ I have excellent hearing  ❑ I hear normally  ❑ I have some difficulty hearing or I do not hear clearly (e.g. when there is background noise)  ❑ I have difficulty hearing things clearly. Often I do not understand what is said. I usually do not take part in conversations because I cannot hear what is said.  ❑ I hear very little  ❑ I am completely deaf. |
| **Q33** | How often do you feel depressed?   \| ❑ Never \| ❑ Almost never \| ❑ Sometimes \| ❑ Often \| ❑ Very often \| ❑ All the time \| \| --- \| --- \| --- \| --- \| --- \| --- \| |
| **Q34** | How happy are you with your close and intimate relationships?  ❑ Very happy  ❑ Generally happy  ❑ Neither happy nor unhappy  ❑ Generally unhappy  ❑ Very unhappy |
| **Q35** | How often did you feel in despair in the last seven days?   \| ❑ Never \| ❑ Occasionally \| ❑ Sometimes \| ❑ Often \| ❑ All the time \| \| --- \| --- \| --- \| --- \| --- \| |

**SECTION 7: SLEEP QUALITY**

We would like to know about the quality of your sleep. Please answer the following questions.

**Pittsburgh Sleep Quality Index (PSQI)**

Instructions: The following questions relate to your usual sleep habits during the past month only. Your answers should indicate the most accurate reply for the majority of days and nights in the past month. Please answer all questions.

| **1.** | During the past month, what time have you usually gone to bed at night? ___________________________________________ | | | | | | |
| --- | --- | --- | --- | --- | --- | --- | --- |
| **2.** | During the past month, how long (in minutes) has it usually taken you to fall asleep each night? _________________________ | | | | | | |
| **3.** | During the past month, what time have you usually gotten up in the morning? _________________________________­______ | | | | | | |
| **4.** | During the past month, how many hours of actual sleep did you get at night? (This may be different than the number of hours you spent in bed.) ____________________________________________________________________________________________ | | | | | | |
| **5.** | | During the past month, how often have you had trouble sleeping because you….. | Not during the past month | Less than once a week | Once or twice a week | Three or more time a week |  |
|  | **a.** | Cannot get to sleep within 30 minutes |  |  |  |  |  |
|  | **b.** | Wake up in the middle of the night or early morning |  |  |  |  |  |
|  | **c.** | Have to get up to use the bathroom |  |  |  |  |  |
|  | **d.** | Cannot breathe comfortably |  |  |  |  |  |
|  | **e.** | Cough or snore loudly |  |  |  |  |  |
|  | **f.** | Feel too cold |  |  |  |  |  |
|  | **g.** | Feel too hot |  |  |  |  |  |
|  | **h.** | Have bad dreams |  |  |  |  |  |
|  | **i.** | Have pain |  |  |  |  |  |
|  | **j.** | Other reasons(s): please describe: |  |  |  |  |  |
| **6.** |  | During the past month, how often have you taken medicine to help you sleep (prescribed or “over the counter”)? |  |  |  |  |  |
| **7.** |  | During the past month, how often have you had trouble staying awake while driving, eating meals, or engaging in social activity? |  |  |  |  |  |

|  | | No problem at all | Only a very slight problem | Somewhat of a problem | A very big problem |
| --- | --- | --- | --- | --- | --- |
| **8**. | During the past month, how much of a problem has it been for you to keep up enough enthusiasm to get things done? |  |  |  |  |

|  | | Very good | Fairly good | Fairly bad | Very bad |
| --- | --- | --- | --- | --- | --- |
| **9**. | During the past month, how would you rate your sleep quality overall? |  |  |  |  |
|  | | No bed partner or room mate | Partner/room mate in other room | Partner in same room but not same bed | Partner in same bed |
| **10**. | Do you have a bed partner or room mate? |  |  |  |  |

|  | | If you have a room mate or bed partner, ask him/her how often in the past month you have had: | Not during the past month | Less than once a week | Once or twice a week | Three or more time a week |
| --- | --- | --- | --- | --- | --- | --- |
|  | **a.** | Loud snoring |  |  |  |  |
|  | **b.** | Long pauses between breaths while asleep |  |  |  |  |
|  | **c.** | Legs twitching or jerking while you sleep |  |  |  |  |
|  | **d.** | Episodes of disorientation or confusion during sleep |  |  |  |  |
|  | **e.** | Other restlessness while you sleep, please describe: |  |  |  |  |

**SECTION 8: BELIEFS ABOUT BACK PAIN**

**Back Beliefs Questionnaire**

We are trying to find out what people think about low back trouble. Please indicate your general views towards back trouble, even if you have never had any.

Completely disagree

Completely agree

1

2

3

4

5

Please answer ALL statements and indicate whether you agree or

disagree with each statement by circling the appropriate number

of the scale.

| Question | | Completely disagree |  |  |  | Completely agree |
| --- | --- | --- | --- | --- | --- | --- |
| **Q1** | There is no real treatment for back trouble | 1 | 2 | 3 | 4 | 5 |
| **Q2** | Back trouble will eventually stop you from working | 1 | 2 | 3 | 4 | 5 |
| **Q3** | Back trouble means periods of pain for the rest of one’s life | 1 | 2 | 3 | 4 | 5 |
| **Q4** | Doctors cannot do anything for back trouble | 1 | 2 | 3 | 4 | 5 |
| **Q5** | A bad back should be exercised | 1 | 2 | 3 | 4 | 5 |
| **Q6** | Back trouble makes everything in life worse | 1 | 2 | 3 | 4 | 5 |
| **Q7** | Surgery is the most effective way to treat back trouble | 1 | 2 | 3 | 4 | 5 |
| **Q8** | Back trouble may mean you end up in a wheelchair | 1 | 2 | 3 | 4 | 5 |
| **Q9** | Alternative treatments are the answer to back trouble | 1 | 2 | 3 | 4 | 5 |
| **Q10** | Back trouble means long periods of time off work | 1 | 2 | 3 | 4 | 5 |
| **Q11** | Medication is the only way of relieving back pain | 1 | 2 | 3 | 4 | 5 |
| **Q12** | Once you have had back trouble there is always a weakness | 1 | 2 | 3 | 4 | 5 |
| **Q13** | Back trouble must be rested | 1 | 2 | 3 | 4 | 5 |
| **Q14** | Later in life back trouble gets progressively worse | 1 | 2 | 3 | 4 | 5 |

**SECTION 9: ATTITUDES TOWARDS PAIN MEDICATIONS**

We would like to better understand people with low back pain’s attitudes towards pain medications. Please answer the following questions.

**Pain Medication Attitudes and Questionnaire (PMAQ-14) Short Form**

The following statements refer to how you feel about pain medications/painkillers.

Please circle the number corresponding to how much you agree with each statement.

|  | | Never true | Almost never true | Seldom true | Often true | Almost always true | Always true |
| --- | --- | --- | --- | --- | --- | --- | --- |
| **Q1** | I am concerned that taking medication for a long time will lead to addiction | 0 | 1 | 2 | 3 | 4 | 5 |
| **Q2** | I worry that my pain medication/s will stop working | 0 | 1 | 2 | 3 | 4 | 5 |
| **Q3** | I am afraid that stopping my pain medication/s will cause me to feel ill | 0 | 1 | 2 | 3 | 4 | 5 |
| **Q4** | I fear that I am becoming an addict | 0 | 1 | 2 | 3 | 4 | 5 |
| **Q5** | I would be unwilling to reduce my pain medication/s | 0 | 1 | 2 | 3 | 4 | 5 |
| **Q6** | I fear that I will eventually run out of pain medication/s that will help with the pain | 0 | 1 | 2 | 3 | 4 | 5 |
| **Q7** | I worry that withdrawal from my pain medication/s will cause me some harm | 0 | 1 | 2 | 3 | 4 | 5 |
| **Q8** | I find it hard to put up with the side effects from my pain medication/s | 0 | 1 | 2 | 3 | 4 | 5 |
| **Q9** | Needing to take medication for my pain embarrasses me | 0 | 1 | 2 | 3 | 4 | 5 |
| **Q10** | I worry what others think about my use of pain medication/s | 0 | 1 | 2 | 3 | 4 | 5 |
| **Q11** | I worry about damage to my internal organs from my pain medication/s | 0 | 1 | 2 | 3 | 4 | 5 |
| **Q12** | I feel confident about my doctor’s management of my pain medication/s | 0 | 1 | 2 | 3 | 4 | 5 |
| **Q13** | I depend on my pain medication/s | 0 | 1 | 2 | 3 | 4 | 5 |
| **Q14** | I feel satisfied with information with doctor gives me about medication/s | 0 | 1 | 2 | 3 | 4 | 5 |

**This is the end of the questionnaire, thank you for participating.**
