# Supplementary material for: Effectiveness of a coordinated support system linking public hospitals to a health coaching service compared with usual care at discharge for patients with chronic low back pain: protocol for a randomised controlled trial
Source: BMC Musculoskelet Disord. 2021 Jul 9;22:611. doi: 10.1186/s12891-021-04479-z (PMC8272287; doi:10.1186/s12891-021-04479-z)
Supplement: Supplementary file 5 — Additional file 5. [file 12891_2021_4479_MOESM5_ESM.docx]

**FORTNIGHTLY FOLLOW-UP QUESTIONNAIRE**

1. Have you experienced low back pain in the last 2 weeks (fortnight)?

❑ No 🡪 skips to Q2

❑ Yes

**PAIN INTENSITY** *(observation: this question will only appear if yes is selected for Q1)*

1a. Over the last 2 weeks (fortnight), what was the average intensity of your low back pain on a scale of 0 (no pain) to 10 (the worst pain imaginable)?

| 0 | 1 | 2 | 3 | 4 | 5 | 6 | 7 | 8 | 9 | 10 |
| --- | --- | --- | --- | --- | --- | --- | --- | --- | --- | --- |
| No pain |  |  |  |  | Moderate Pain |  |  |  |  | Worst possible pain |

**PAIN FREQUENCY** *(observation: this question will only appear if yes is selected for Q1)*

1b. Over the last 2 weeks (fortnight), how many days did you experience low back pain?_____________________

**USE OF CARE OF TREATMENTS FOR LOW BACK PAIN**

**Hospital, Medical and Health Services for Low Back Pain**

**2.** Over the last 2 weeks (fortnight), did you seek care from any medical or health services for your low back pain?

| ❑ I did not seek care from any health professionals or health services 🡪 *skip to Q3* | | |
| --- | --- | --- |
| ❑ Surgery | **i**. What type of surgery?  ❑ Microdiscectomy ❑ Discectomy ❑ Laminectomy  ❑ Decompression ❑ Fusion ❑ Unsure  ❑ Other, please specify  **ii**. What type of hospital did you receive treatment? ❑ Private Hospital ❑ Public Hospital  **iii**. How much did this surgery cost (out of pocket) in total? | |
| ❑ Emergency department visit | **iv**. How many days did you spend at the hospital in total? | |
| ❑ Nursing | **v**. Where did you encounter a nurse?  ❑ Hospital ❑ GP/medical practice ❑ Other, please specify  **vi**. How many visits/sessions?  **vii**. How much did these visits/sessions cost (out of pocket) in total over the last 2 weeks? | |
| ❑ Imaging/Scans | **viii**. Please indicate which type(s): | |
|  | ❑ X-ray  ❑ CT scan  ❑ MRI | ❑ Ultrasound  ❑ Nerve conduction studies  ❑ Other, please specify |
|  | **iv**. How much did these scans cost (out of pocket) in total over the last 2 weeks? | |
| ❑ General practitioner (GP) | **x**. How many visits/sessions?  ix. How much did these visits/sessions cost (out of pocket) in total over the last 2 weeks? | |
| ❑ Pharmacist | **xi**. How many visits to a pharmacist?  **xii**. How much did these visits/sessions cost (out of pocket) in total over the last 2 weeks? | |
| ❑ Physiotherapist | **xiii**. Where did you visit a physiotherapist?  ❑ Public hospital  ❑ Private hospital  ❑ Private clinic  **xiv**. How many visits/sessions?  **xv**. How much did these visits/sessions cost (out of pocket) in total over the last 2 weeks? | |
| ❑ Chiropractic | **xvi**. How many visits/sessions?  **xvii**. How much did these visits/sessions cost (out of pocket) in total over the last 2 weeks? | |
| ❑ Exercise physiologist | **xviii**. Where did you visit an exercise physiologist?  ❑ Public hospital ❑ Private hospital ❑ Private clinic  **xix**. How many visits/sessions?  **xx**. How much did these visits/sessions cost (out of pocket) in total over the last 2 weeks? | |
| ❑ Massage therapist | **xxi**. How many visits/sessions?  **xxii**. How much did these visits/sessions cost (out of pocket) in total over the last 2 weeks? | |
| ❑ Psychiatrist, Psychologist, or Counsellor | **xxiii**. Where did you visit a psychiatrist, psychologist, or counsellor?  ❑ Public hospital ❑ Private hospital ❑ Private clinic  **xxiv**. How many visits/sessions?  **xxv**. How much did these visits/sessions cost (out of pocket) in total over the last 2 weeks? | |
| ❑ Health coaching | **xxvi**. How many visits/sessions?  **xxvii**. How much did these visits/sessions cost (out of pocket) in total over the last 2 weeks? | |
| ❑ Natural therapies (e.g. acupuncture) | **xxviii**. How many visits/sessions?  **xxvix**. How much did these visits/sessions cost (out of pocket) in total over the last 2 weeks? | |
| ❑ Osteopath | **xxx**. How many visits/sessions?  **xxxi**. How much did these visits/sessions cost (out of pocket) in total over the last 2 weeks? | |
| ❑ Specialist | **xxxii**. Please indicate which type(s):  ❑ Orthopaedic surgeon ❑ Pain physician  ❑ Rheumatologist ❑ Neurologist  ❑ Other, please specify:  *(Observation*: *the following questions will only appear if the participant has selected any of the specialist options.)*  **xxxiii**. How many appointments?  **xxxiv**. How much did these visits/sessions cost (out of pocket) in total over the last 2 weeks? | |
| ❑ Other, please specify:  _______________________________ | **xxxv.** How many treatment sessions/appointments?  **xxix.** In total over the last 2 weeks, how much did these visits/sessions cost you (out of pocket)? | |

(*Observation*: *this question will only appear if the participant has selected any option except “I did not seek care”*

**Traveling Time**

**2a.** In total, how much time in total did you spend travelling to and from all the health professional/services you listed above, in the last 2 weeks (fortnight)? This includes time spent travelling by car, catching public transport or walking etc. ­________________ hours :________________minutes

**Prescribed Medications for Low Back Pain**

1. Over the last 2 weeks (fortnight), did a medical or health practitioner (e.g., GP, pharmacist, specialist) **prescribe** you any medications for your low back pain?

❑ No

❑ Yes

**Medication Use for Low Back Pain**

1. Over the last 2 weeks (fortnight), did you **use** any pain medications for low back pain in the last 2 weeks (fortnight)?

❑ No 🡪 skips to Q5

❑ Yes

**4a**. Please indicate which pain medications and answer any relevant questions:

|  | *(Observation*: *the following questions in this column will only appear if the participant has selected a given medication)* |
| --- | --- |
| ❑ Paracetamol (e.g., Panadol) | **i.** Was this medication prescribed to you by a medical or health practitioner (e.g., GP, pharmacist, specialist)? Yes / No  **ii.** How many days did you take paracetamol for your low back pain within the last 2 weeks (fortnight)?  **iii.** On the days you took paracetamol for your low back pain, what was the average number of tablets you took per day?  **iv.** What was the dosage (milligrams per tablet)? |
| ❑ NSAIDs (e.g. neurofen, ibuprofen) | **i.** Was this medication prescribed to you by a medical or health practitioner (e.g., GP, pharmacist, specialist)? Yes / No  **ii.** How many days did you take NSAIDs tablets for your low back pain within the last 2 weeks (fortnight)?  **iii.** On the days you took NSAIDs for your low back pain, what was the average number of NSAID tablets you took per day?  **iv.** What was the dosage (milligrams per tablet)? |
| ❑ Opioids (e.g. codeine, oxycodone, morphine, fentanyl, hydrocodone) | **i.** Was this medication prescribed to you by a medical or health practitioner (e.g., GP, pharmacist, specialist)? Yes / No  **ii.** How many days did you take opioid tablets for your low back pain within the last 2 weeks (fortnight)?  **iii.** On the days you took opioids for your low back pain, what was the average number of opioid tablets you took per day?  **iv.** What was the dosage (milligrams per tablet)? |
| ❑ Other, please specify: _______________________ | **i.** Was this medication prescribed to you by a medical or health practitioner (e.g., GP, pharmacist, specialist)? Yes / No  **ii.** How was the pain medication used? ❑ Tablet ❑ Patch ❑ Other  If tablet:  **iii.** How many days did you take this pain medications for this low back pain within the last 2 weeks (fortnight)?  **iv.** On the days you took this pain medication for your low back pain, what was the average number of tablets you took per day?  **v.** What was the dosage (milligrams per tablet)?  If patch:  **vi.** What was the dosage (milligrams per patch)?  **vii**. How often did you use a patch (1 patch per week)?  If other:  **viii.** How did you use the medication (e.g., injection, apply to skin)?  **ix.** What was the dosage?  **x**. How often did you use the medication? |

**Other Self-Management Techniques**

**5.** Excluding pain medications, did you use any other self-management behaviours **specifically to manage your low back pain** in the last 2 weeks (fortnight)?

❑ No 🡪 *end of questionnaire*

❑ Massage (i.e., not from a professional)

❑ Heat packs or hot shower

❑ Brace or support strapping/tape

❑ Topical creams/gels (e.g., Voltaren)

❑ Physical activity and exercise

❑ Relaxation, meditation, or mindfulness techniques

❑ Walking aids (e.g., crutches, walking stick)

❑ Other, please specify ____________________

*Observation*: *the following questions will only appear if the participant has selected any of the options except “no”*

**5a.** Did you purchase any of the items that you selected in the previous question within the last 2 weeks (fortnight)?

❑ No, or not relevant 🡪 *end of questionnaire*

❑ Yes

**5b.** Please list which items _______________________

**5c.** How much did the item(s) cost you in total?______________

**5d.** How much time did you spend travelling to and from the store to purchase these items?

(HH:MM) ________________ hours :________________minutes

**Thank you for completing the questionnaire.**

**You will receive the next questionnaire in approximately 2 weeks.**
