# Supplementary material for: Effectiveness of a coordinated support system linking public hospitals to a health coaching service compared with usual care at discharge for patients with chronic low back pain: protocol for a randomised controlled trial
Source: BMC Musculoskelet Disord. 2021 Jul 9;22:611. doi: 10.1186/s12891-021-04479-z (PMC8272287; doi:10.1186/s12891-021-04479-z)
Supplement: Supplementary file 6 — Additional file 6. [file 12891_2021_4479_MOESM6_ESM.docx]

*
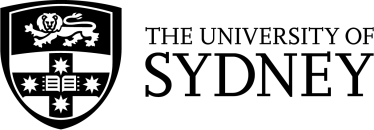
*

**Participant Information Sheet and Consent Form**

**Health/Social Science Research**

| **Title** | The Get Healthy Coaching Service® to reduce the burden of low back pain: effectiveness, cost effectiveness, and scalability |
| --- | --- |
| **Short Title** | Get Back to Healthy project |
| **Protocol Number** | 6 |
| **Project Sponsor** | University of Sydney |
| **Coordinating Principal Investigator** | Professor Paulo Ferreira |
| **Investigator(s)** | Prof Manuela Ferreira, A/Prof Milena Simic, Ms Dragana Ceprnja, Ms Katherine Maka, Dr Mark Halliday, Ms Emma Ho, Mr Thomas Patterson |
| **Location** | Westmead Hospital |

**Part 1 What does my participation involve?**

**1 Introduction**

You are invited to take part in this research study.

This Participant Information Sheet/Consent Form tells you information about the research project. It explains what taking part in the study involves. Knowing what is involved will help you decide if you want to take part in the research.

Please read this information carefully and ask questions about anything that you don’t understand or want to know more about. Before deciding whether or not to take part, you might want to talk about it with a relative, friend or local health worker.

Your participation in this study is completely voluntary and there will be no cost to you. If you do not want to take part in this study you do not have to. You should feel under no obligation to participate in this study. Choosing not to take part in this study will not affect your current and future medical care in any way.

If you decide you want to take part in the research project, you will be asked to sign the consent section. By signing it you are telling us that you:

• Understand what you have read

• Consent (given permission) to take part in the research project

• Consent to be involved in the research described

• Consent to the use of your personal and health information as described.

You will be given a copy of this Participant Information and Consent Form to keep.

**2 What is the purpose of this research?**

The purpose of this research is to test whether a ‘***back pain support system****’* can help people with low back pain maintain improvements in their symptoms and physical activity levels after finishing a course of physiotherapy treatment at Westmead Hospital. The study will also measure whether the ***back pain support system*** changes people’s use of hospital, medical and health services for low back pain. The back pain support system will involve a health coaching program delivered over the phone. The program is run by the Get Healthy Service®, which is part of NSW Health. The program will involve having a personal health coach to help support you to achieve healthy lifestyle goals that are important to you. The back pain support system will be compared to the usual care people finishing physiotherapy treatment receive, which may include advice, education and exercises, to see if it better helps and supports you to manage your back pain after finishing physiotherapy treatment at the hospital.

This research has been initiated by the researcher Professor Paulo Ferreira from the University of Sydney. The results of this research will be used by Ms Emma Ho to obtain a Doctor of Philosophy (Health Sciences) degree.

**3 What does participation in this research involve?**

You will be participating in a randomised controlled research project. Sometimes we do not know which treatment is best for treating a condition. To find out we need to compare different treatments. We put people into groups and give each group a different treatment. The results are compared to see if one is better. To try to make sure the groups are the same, each participant is put into a group by chance (random).

You have been invited because you have low back pain for more than 3 months, you are older than 18 years of age, and you have recently finished (or are close to finishing) physiotherapy treatment for your low back pain at the Outpatient Physiotherapy Department of Westmead Hospital. After reading this information sheet, speak with your physiotherapist or contact the research team if you are interested in the study or have any questions. The study will take one year to complete.

Participation in the research will involve the following:

When you are close to finishing your hospital physiotherapy program, your physiotherapist will introduce the study to you. If you are interested, your physiotherapist will ask for your permission to give your contact details (phone number) to the research team.

The research team will call you to give you more information about the study. You will be given the study information package to read and discuss with your family, friends and GP (if you wish). After approximately one week, the research team will call you again to confirm if you are still interested in the study. You do not have to take part in the study. If you do not wish to participate, your care at the hospital will not be affected.

If you are interested in taking part in the study, the research team will organise a time to discuss the study Participant Information Sheet with you. They will answer any questions you have about the study. If you agree to participate, you will be asked to sign the study consent form. You can choose to sign the consent form online (via an online link) or in person at the hospital. If you choose to sign the consent form online, a research team member will speak with you via phone call or videoconference to give you support. The study consent form must be signed **before** any further study procedures occur. After signing the consent form, you will be immediately assigned a **unique participant study code**. This participant code will be used on all study documents to protect your privacy.

The Participant Information Sheet will clearly explain how and when your contact information will be used. On the consent form, you will be asked to indicate **your preferred method(s)** for the research team to contact you (i.e. phone call, SMS, email, mailing address). You will be asked to provide these contact details on the consent form. Your contact information will be stored in a secure, password-protected server hosted by the University of Sydney. Only the research team will have access to your contact information. A copy of the main study consent form is attached at the end of the Participant Information Sheet.

If you decide to take part in the research project, you will be asked to complete a questionnaire asking about your low back pain and medical history. The questionnaire will assess if you are eligible to take part in the study. Completing the questionnaire will take approximately 5-8 minutes. If the screening questionnaire shows that you meet the requirements, then you will be able to start the research project. If the screening questionnaire shows that you cannot be in the research project, the research coordinator will discuss other options with you.

For safety reasons, the research team *may* request you seek approval from your local doctor (GP) before joining the study. If so, you will be given a form for your local doctor to sign. You will need to return the signed form to the research team **before** any further study procedures occur.

Once the research team confirms you are both suitable and safe to join the study, you will be ***enrolled*** into the study.

The research team will request additional permission to access your Medicare (MBS) and Medicine (PBS) data. Medicare collects information on your doctor visits and associated costs. PBS collects information on the prescription medications you purchase at pharmacies. This data will provide valuable information about your use of hospital, medical and health services, and medications. You will receive a **separate** MBS/PBS Information Sheet and Consent Form that explains this information in detail. If you agree, you will be asked to sign a separate MBS/PBS consent form (hard-copy version). You can choose to sign the form in person at the hospital, or you can request for the research team to post the form to your mailing address.

Once you are enrolled in the study, you will be invited to complete an assessment with a member of the research team. You can choose to complete this initial assessment in person at the hospital or online (with phone or videoconference support from the research team).

There are three parts involved in the initial assessment:

1. Completing an electronic questionnaire: You will be asked to complete a **questionnaire** about your height and weight, education levels, medical history, low back pain symptoms, use of treatments, sleep, attitudes towards pain medications and beliefs about back pain. It will take approximately 35 minutes to complete. If you prefer to complete the initial assessment online, you will be emailed a link to the questionnaire.

2. Wearing a physical activity device: You will be asked to wear a **physical activity device**, similar to a Fitbit. The device records information about how active you are (e.g. number of steps). The device will be attached to your right leg using 3 pieces of tape. You will need to wear the device for 7 days in a row. You can continue most of your normal activities during this time.

You will also be given a paper **logbook** to record any physical activity or exercise you complete whilst wearing the device. You will receive reminders (via your preferred contact method) to return the device and logbook back to the research team at the end of 7 days. We will give you a pre-paid envelope to return these items to the research team.

If you complete the initial assessment in person at the hospital, the research team will help you to attach the device to your leg. If you compete initial assessment online, the device and paper logbook will be posted to your mailing address. You will receive instructions on how to attach the device to your leg by yourself or with help from a family/friend. The research team will be available via phone call or video conference to help you if needed.

3. Completing a weekly diary: The research team will also give you a paper **weekly diary** to record any discomfort or incidents that may occur during the study. The research team will explain how to use this diary. The diary will track your safety every week during the first 6 months of the study. You will receive reminders to complete the diary (via your preferred contact method). After 6 months, you will need to return the diary to the research team.

After completing the initial assessment, each participant will be put into a study group by chance (random). There are **two *possible*** study groups involved in this study**.** The groups are either the: (1) Usual Care Control Group or (2) Back Pain Support System Group. The research team will use a computer software program to **randomly select** which study group you will join.

You have a **50% chance** of being put in either of the following two groups:

Usual Care Control Group (Study Group 1):

- If you are randomly put in the usual care group, this means that you will be asked to continue with the **usual care program** that is recommended by your physiotherapist.
- This may include a program of advice, education and exercises to complete at home or in your local community.
- You ***will not*** be asked to participate in the health coaching sessions. However, you will be offered the opportunity to participate in the Get Healthy Service® ***after*** completing your 12-month follow-up assessment.

Back Pain Support System Group (Study Group 2):

- If you are randomly put in the back pain support system group, this means you will be asked to continue with the **usual care program** that is recommended by your physiotherapist. In addition, you will be asked to take part in a **health coaching program.**
- The **health coaching program** will be delivered by the Get Healthy Service®. The research team will need to provide your personal details (name, date of birth, phone number) to the service so they can deliver the health coaching sessions. If you give permission, the research team will also provide the Get Healthy Service® with your email address and postal address. The Get Healthy Service® is funded by the NSW Ministry of Health and will store your personal information securely and confidentially. If you needed additional medical clearance before joining the study, the Get Healthy Service® may ask for a copy of your medical referral form. If so, the research team will send a copy of your form via a secure program used by the NSW Ministry of Health.
- You will receive up to 10 health coaching sessions over 6 months. All sessions will be delivered **over the phone** by a trained health coach. You can decide how often and how many sessions you will take part in.
- In the first session, the health coach will help you set goals to increase your physical activity levels, as well as any other health-related goals if you wish to work on (e.g. improve diet, lose weight, reduce alcohol consumption).
- Your health coach will support you and monitor your progress during the program.
- After completing the program, you have the additional option to enrol into further health coaching sessions or join a free SMS program for another 6 months (called the *Get Healthy Stay Healthy* SMS program). This program will send you automatic SMS messages with tips to stay on track with your goals. If you choose this option, your health coach may contact you periodically to check on your progress.

All participants in both study groups will be asked to take part in the follow-up data collection. Follow-up data collection will continue for **one year** from the start of the study.

It will involve:

1. Completing a fortnightly questionnaire: Every fortnight (2 weeks), you will receive a link to a **brief online** **questionnaire**. We will send you the link via SMS or email, depending on your preferred contact method. The questionnaire will ask if you experienced low back pain in the past fortnight. You may be asked extra questions related to the pain intensity and whether you used any care or treatment for the pain. It will take roughly 1 minute to complete the questions (maximum 5 minutes). Occasionally, you may receive reminders to complete the questionnaires.

2. Additional assessments at 6 and 12 months:

(1) **An online** **questionnaire**: At 6 months and 12 months after joining the study, you will be asked to complete an online questionnaire. The questionnaire will be similar to the initial assessment questionnaire, but with less questions. You will receive a link to the online questionnaire (via SMS or email). It will take roughly 25 minutes to complete.

(2) At 6 months after joining the study (not at 12 months), you will also be asked to wear the **physical activity device** and complete the **logbook** again for 7 days. A package containing the device and logbook will be posted to your mailing address. Before sending the package, the research team will contact you (via your preferred contact method) to confirm you are available to receive it (e.g. not away on holidays).

At 3, 6, and 9 months into the study, the research team will also briefly contact you, via your preferred contact method. The research team member will ask if you have any concerns about being in the study. At the end of the study (12 months), you *may* also be asked to take part in an **interview** (approximately one hour). You may be asked questions about your experiences during the study.

**4 Other relevant information about the research project**

This research project has been designed to make sure the researchers interpret the results in a fair and appropriate way. The research project has been designed to prevent study staff or participants jumping to conclusions.

There are no additional costs associated with participating in this research project, nor will you be paid. If we ask you to take part in the health coaching program, it will be provided to you free of charge.

**5 Do I have to take part in this research project?**

Participation in any research project is voluntary. If you do not wish to take part, you do not have to. If you decide to take part and later change your mind, you are free to withdraw from the project at any stage.

If you decide to take part, you will be given this ‘**Participant Information and Consent Form’** to sign. You will be given a copy to keep. Your decision whether to take part or not to take part, or to take part and then withdraw, will not affect your routine treatment, your relationship with those treating you or your relationship with Westmead Hospital.

**6 What are the alternatives to participation?**

You do not have to take part in this research project to receive treatment at this hospital. You can continue with the usual care provided by your physiotherapist, without participating in the study. If you do not wish to take part in the study, your physiotherapist or the research team can discuss other options with you.

**7 What are the possible benefits of taking part?**

We cannot guarantee or promise that you will receive any benefits from this research. However, possible benefits may include increased support for physical activity participation and reduced pain and disability.

**8 What are the possible risks and disadvantages of taking part?**

You may feel that some of the questions we ask are stressful or upsetting. If you do not wish to answer a question, you may skip it and go to the next question, or you may just stop the questions. If you become upset or distressed as a result of your participation in the research project, the research team will be able to arrange for counselling or other appropriate support. Any counselling or support will be provided by qualified staff who are not members of the research team. This counselling will be provided free of charge.

There is a small risk of some muscle soreness from participating in the study. If you do experience any muscle soreness during the study, it will most likely be from taking part in new types of activities or exercising more than usual. We expect that any soreness would settle quickly after a few days.

Please take care when doing exercise. If a serious incident occurs, please complete your weekly diary immediately and contact the research team as soon as possible. Please call 000 if it is an emergency.

**9 Can I have other treatments during this research period?**

Whilst taking part in the study, you will still be able to take all medications or treatments you have been taking for your low back pain or for other reasons.

**10 What if I withdraw from this research project?**

If you do consent to participate, you may withdraw at any time. If you decide to withdraw from the project, please notify a member of the research team before you withdraw. A member of the research team will inform you if there are any special requirements linked to withdrawing. If you do withdraw, you will be asked to complete and sign a ‘Withdrawal of Consent’ form; this will be provided to you by the research team.

If you decide to leave the research project, the researchers will not collect additional personal information from you, although personal information already collected will be retained to ensure that the results of the research project can be measured properly and to comply with law.

You should be aware that data collected up to the time you withdraw will form part of the research project results. If you do not want your data to be included, you must tell the researchers when you withdraw from the research project.

**11 Could this research project be stopped unexpectedly?**

This research project has no reasons to be stopped unexpectedly.

**12 What happens when the research project ends?**

At the end of the study, the research team will send you a summary of the study findings if you wish. You will be asked to indicate this on the consent form and provide your email address if so.

For participants in the usual care group only: After completing the 12-month assessment, participants in the usual care control group will be contacted by the research team via phone call. The research team will confirm whether you have enrolled into any of the Get Healthy Service® programs and will offer you the opportunity to join any of the Get Healthy Service® health coaching programs if you wish.

**Part 2 How is the research project being conducted?**

**13 What will happen to information about me?**

By signing the consent form, you give permission for the research team to collect and use personal information about you for the research project. Any information obtained in connection with this research project that can identify you will remain confidential and stored securely. Your information will only be used for the purpose of this research project and it will only be disclosed with your permission, except as required by law. Your personal contact details for the study which will be collected from you via the consent form (e.g. your phone number, email address and mailing address) will only be used for study procedures, such as sending you study documents, equipment and reminders. Any data collected from you that may identify you will be stored on a secure, confidential, password-protected online data collection software called REDCap (hosted by the University of Sydney). Only approved members of the research team will have access to your personal contact details.

To protect your privacy, you will be given a unique participant code so that your name and details are not used on study documents. The research team will collect information about your medical history, symptoms and treatments, and general health (e.g. sleep quality) from questionnaires, diaries and an activity device. Your data will be stored on a secure, password-protected REDCap data collection program. Your data will be stored separate to any personal information about you. No-one can identify you from your data, except for members of the research team who have special approved access.

Only approved members of the research team, the Human Research Ethics Committee (HREC) for monitoring purposes, persons monitoring the conduct of the study on behalf of the Project Sponsor (i.e. chief principal investigator, principal investigator, clinical trial coordinator, research staff), or regulatory bodies (including the Therapeutic Goods Administration) will have access to your details.

Information about you may be obtained from your health records held at this and other health organisations for the purpose of this research. This may include linking to your hospital and Medicare and Prescribed Medicines (MBS/PBS) data. By signing the study consent form and separate MBS/PBS consent form, you give permission for the research team to access your health records and MBS/PBS data, if they are relevant to your participation in this research project.

Your health records and any information collected about you that is relevant to the research project may be reviewed for verifying study procedures and data. This review may be done by the relevant authorities and authorised representatives of the Sponsor (University of Sydney), the institution relevant to this Participant Information Sheet (Western Sydney Local Health District (WSLHD) HREC) or as required by law. By signing both the study and MBS/PBS consent forms, you authorise release of, or access to, this confidential information to the relevant research personnel and regulatory authorities as noted above.

It is expected that the results of this research project will be published and/or presented in a variety of forums and peer reviewed journals. The results may also be used in a PhD thesis at the University of Sydney. You will not be able to be identified in any publications and/or presentations, except with your permission. Your data may be used for extended (related) research projects. Separately, your MBS/PBS data will be stored on a secure, confidential, password protected network server hosted by the University of Sydney. Once the research team links your study data to your MBS/PBS data, the research team will remove any identifying information (e.g. personal details) from your MBS/PBS data. Your MBS/PBS data will not be used in any future or unspecified research outside of the approved study.

Information about your participation in this research project may be recorded in your health records.

In accordance with relevant Australian and/or NSW privacy and other relevant laws, you have the right to request access to the information about you that is collected and stored by the research team. You also have the right to request that any information with which you disagree be corrected. Please inform the research team member named at the end of this document if you would like to access your information.

After removing any identifying information from the data, the research team will keep your study data archived on the secure University of Sydney’s server for 15 years. This is consistent with clinical trial recommendations outlined in section 2.1.1 of the National Health and Medical Research Council’s “Australian Code for the Responsible Conduct of Research”. Your MBS/PBS will undergo a different process. According to the requirements of Services Australia, your MBS/PBS data will be destroyed 15 years after results of the project are published.

**14 Complaints and compensation**

If you suffer any injuries or complications as a result of this research project, you should contact the research team as soon as possible and you will be assisted with arranging appropriate medical treatment. In the event of loss or injury, there will be no special compensation agreements. In the event of loss or injury due to someone’s negligence, you may have grounds for legal action but may have to pay for the expenses. If you wish you complain or have concerns about any aspects of how you have been treated during the study, you are advised to contact the WSLHD HREC. If you are eligible for Medicare, you can receive any medical treatment required to treat the injury or complication, free of charge, as a public patient in any Australian public hospital.

**15 Who is organising and funding the research?**

This research project is being conducted by the University of Sydney and will be led by Professor Paulo Ferreira. Associated researchers are from WSLHD and The University of Sydney and have experience in conducting research projects. The project also involves a partnership with the Get Healthy Service®, which is funded and managed by the NSW Government (Ministry of Health) and is free of charge to participants. This project is funded by an Allied Health Kickstarter Grant from WSLHD and a Partnership Grant from the National Health and Medical Research Council.

**16 Who has reviewed the research project?**

All research in Australia involving humans is reviewed by an independent group of people called a Human Research Ethics Committee (HREC). The ethical aspects of this research project have been approved by the HREC of WSLHD. This project will be carried out according to the *National Statement on Ethical Conduct in Human Research (2007)*. This statement has been developed to protect the interests of people who agree to participate in human research studies.

**17 Further information and who to contact**

The person you may need to contact will depend on the nature of your query.

If you want any further information concerning this project or if you have any medical problems which may be related to your involvement in the project (for example, any side effects), you can contact the principal study doctor (Lead Investigator) or any of the following people:

**Lead Investigator Contact Details**

| Name | Professor Paulo Ferreira |
| --- | --- |
| Position | Chief investigator |
| Telephone | (02) 8627 7062 |
| Email | paulo.ferreira@sydney.edu.au |

**Central Research Team Contact Details**

| Name | Ms Emma Ho |
| --- | --- |
| Position | Central research team staff member |
| Telephone | 02 9114 4808 |
| Email | getbacktohealthy.study@sydney.edu.au |

For matters relating to research at the site at which you are participating, the details of the local site complaints person are:

**Complaints contact person**

| Name | Patient Experience Unit |
| --- | --- |
| Position | Patient Experience Unit |
| Telephone | (02) 8890 7014 |
| Email | Wslhd-westmead-feedback@health.nsw.gov.au |

If you have any complaints about any aspect of the project, the way it is being conducted or any questions about being a research participant in general, then you can contact:

**Reviewing HREC approving this research**

| Reviewing HREC | WSLHD Human Research Ethics Committee |
| --- | --- |
| Telephone | (02) 8890 9007 |
| Email | Wslhd-researchoffice@health.nsw.gov.au |

**Local HREC Office contact**

| Position | Research Governance Manager |
| --- | --- |
| Telephone | (02) 8890 9007 |
| Email | [wslhd-researchoffice@health.nsw.gov.au](mailto:wslhd-researchoffice@health.nsw.gov.au) |

*
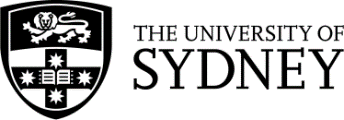
*

**Participant Consent Form**

| **Title** | *The Get Healthy Coaching Service® to reduce the burden of low back pain: effectiveness, cost effectiveness, and scalability* |
| --- | --- |
| **Short Title** | Get Back to Healthy project |
| **Protocol Number** | 6 |
| **Project Sponsor** | University of Sydney |
| **Co-ordinating Principal Investigator** | Professor Paulo Ferreira |
| **Investigator(s)** | Prof Manuela Ferreira, A/Prof Milena Simic, Ms Dragana Ceprnja, Ms Katherine Maka, Dr Mark Halliday, Ms Emma Ho, Mr Thomas Patterson |
| **Location** | Westmead Hospital |

**Declaration by Participant**

1. I have read the Participant Information Sheet or someone has read it to me in a language that I understand.
2. I understand the purposes, procedures and risks of the research described in the project.
3. I have had an opportunity to ask questions and I am satisfied with the answers I have received.
4. I give permission for my doctors, other health professionals, or hospitals outside this hospital to release information to the University of Sydney concerning my disease and treatment for the purposes of this project. I understand that such information will remain confidential.
5. I freely agree to participate in this research project as described and understand that I am free to withdraw at any time during the project without affecting my future care.
6. I acknowledge that regulatory authorities may have access to my medical records specifically related to this project to monitor the research in which I am agreeing to participate. However, I understand my identity will not be disclosed to anyone else or in publications or presentations.
7. I understand that, if I decide to discontinue the study treatment, I may be asked to attend follow-up visits to allow collection of information regarding my health status. Alternatively, a member of the research team may request my permission to obtain access to my medical records for collection of follow-up information for the purposes of research and analysis.
8. I give permission for the research team to use and confidentially store my personal contact information, specifically for the purposes of conducting study procedures.
9. I understand that if I am put in the back pain support group, my personal details (name, date of birth, phone number) and medical referral form (if required) will be sent securely to the Get Healthy Service®, who will store my information confidentially.
10. I understand that I will be given a signed copy of this document to keep.

My best contact details are (please also tick your preferred contact method(s)):

Mobile phone number: ____________________________________ (mobile)

Home phone number: ______________________________________(home)

Email address: _________________________________________________

Mailing address: ________________________________________________

Please indicate:  I wish to receive feedback from my participation at the end of the study;

I wish to receive a summary of the study findings at the end of the study.

| Name of Participant (PRINT) _____________________________________________________  Signature ____________________________ Date _________________________________ |
| --- |

**Declaration by Researcher^†^**

I have given a verbal explanation of the research project, its procedures and risks and I believe that the participant has understood that explanation.

| Name of Researcher^†^ (PRINT) ____________________________________________________  Signature ____________________________ Date _________________________________ |
| --- |

† An appropriately qualified member of the research team must provide the explanation of, and information concerning, the research project. Note: All parties signing the consent section must date their own signature.

*
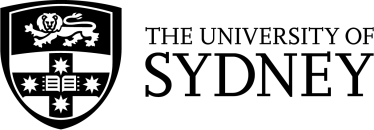
*

**Form for Withdrawal of Participation**

| **Title** | *The Get Healthy Coaching Service® to reduce the burden of low back pain: effectiveness, cost effectiveness, and scalability* |
| --- | --- |
| **Short Title** | Get Back to Healthy project |
| **Protocol Number** | 6  2 |
| **Project Sponsor** | University of Sydney |
| **Co-ordinating Principal Investigator** | Professor Paulo Ferreira |
| **Investigator(s)** | Prof Manuela Ferreira, A/Prof Milena Simic, Ms Dragana Ceprnja, Ms Katherine Maka, Dr Mark Halliday, Ms Emma Ho, Mr Thomas Patterson |
| **Location** | Westmead Hospital |

**Declaration by Participant**

I wish to withdraw from participation in the above research project. I understand that:

1. withdrawal will not affect my routine care, or my relationships with the researchers or Westmead Hospital;
2. no further information about me will be collected for the study from the withdrawal date;
3. information about me that has already been analysed and/or included in a publication by the study, may not be able to be destroyed.

|  | | | | | | | |
| --- | --- | --- | --- | --- | --- | --- | --- |
|  | Name of Participant (please print) | |  | |  |  |  |
|  | | | | | | | |
|  | Signature |  | | Date | |  |  |
|  | | | | | | | |

In the event that the participant’s decision to withdraw is communicated verbally, the Senior Researcher must provide a description of the circumstances below.

|  |
| --- |

**Declaration by Researcher^†^**

I have given a verbal explanation of the implications of withdrawal from the research project and I believe that the participant has understood that explanation.

|  | | | | | | |
| --- | --- | --- | --- | --- | --- | --- |
|  | Name of Researcher (please print) | |  | | |  |
|  | | | | | |  |
|  | Signature |  | | Date |  |  |
|  | | | | | | |

^†^ An appropriately qualified member of the research team must provide information concerning withdrawal from the research project.

Note: All parties signing the withdrawal of participation must date their own signature.

**This form should be forwarded by email to: getbacktohealthy.study@sydney.edu.au**

**Alternatively, this form can be posted to: Professor Paulo Ferreira, School of Physiotherapy, Level 7, Western Avenue, D18 – Susan Wakil Health Building, The University of Sydney, NSW, 2006.**
