# Supplementary material for: Effectiveness of a coordinated support system linking public hospitals to a health coaching service compared with usual care at discharge for patients with chronic low back pain: protocol for a randomised controlled trial
Source: BMC Musculoskelet Disord. 2021 Jul 9;22:611. doi: 10.1186/s12891-021-04479-z (PMC8272287; doi:10.1186/s12891-021-04479-z)
Supplement: Supplementary file 7 — Additional file 7. [file 12891_2021_4479_MOESM7_ESM.docx]

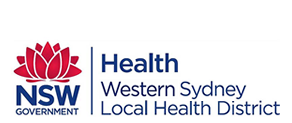
*
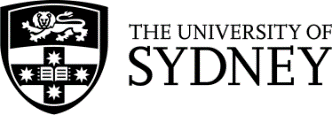
***GET BACK TO HEALTHY STUDY**

**ACTIVITY DEVICE INSTRUCTIONS**

Thank you for participating in the study. At the start, and at 6 months into the study, we would like you to **wear an activity device** (similar to a Fitbit) for **7 days in a row**. The device measures how active you are. During the 7 days, **please also complete a logbook entry every day (page 3).**

If you need help to wear the device, you can ask a family member or friend. The research team can also help you via phone call or video conference.

**Please contact the research team** if you need help, or have any problems with the device or logbook:

Phone: 02 9114 4808 or Email: **getbacktohealthy.study@sydney.edu.au**

**Equipment**

You will receive **3 different sizes** of tape (including spares). Each tape is labelled with a number.

- Tape 1: 1cm x 2cm, smallest size, double-sided white colour.
- Tape 2: 3cm x 5cm, medium size, white colour.
- Tape 3: 8cm x 10cm, largest size, clear colour.

We have also given you an alcohol wipe to clean your skin.

**What can (or can’t) I do whilst wearing the activity device?**

- You can continue your normal daily activities, such as working, exercise (including walking and water sports), and showering, whilst wearing the activity device.
- The activity device is waterproof to 1.5 meters, so you can swim or exercise in the water while having it on your thigh. However, **you cannot dive deeper than 1.5 meters**.
- **Please do not wear it in the ocean** in case if falls off.
- Also, **please take care of the tape** and do not rub it strongly with a towel after taking a shower. If you need to change the tape, please follow the instructions on page 1.

**Where will the activity device be attached?**


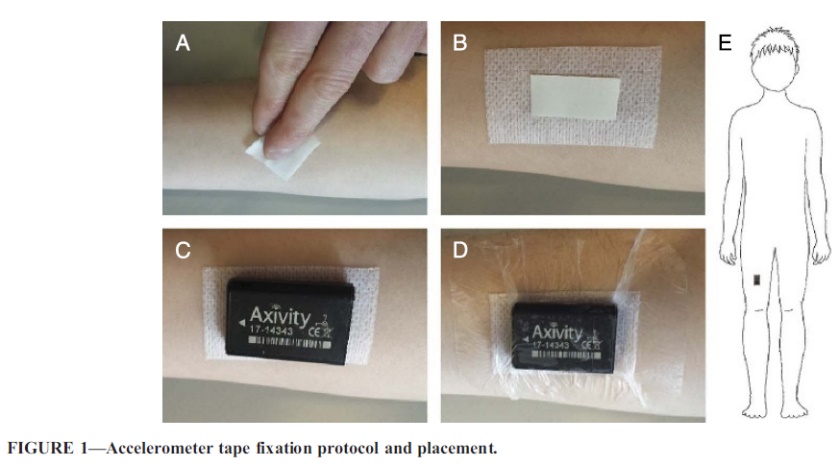
The activity device will be attached to your **RIGHT thigh**. The device should be roughly **2/3rd** of the distance from your right thigh to your knee (e.g. closer to your knee).


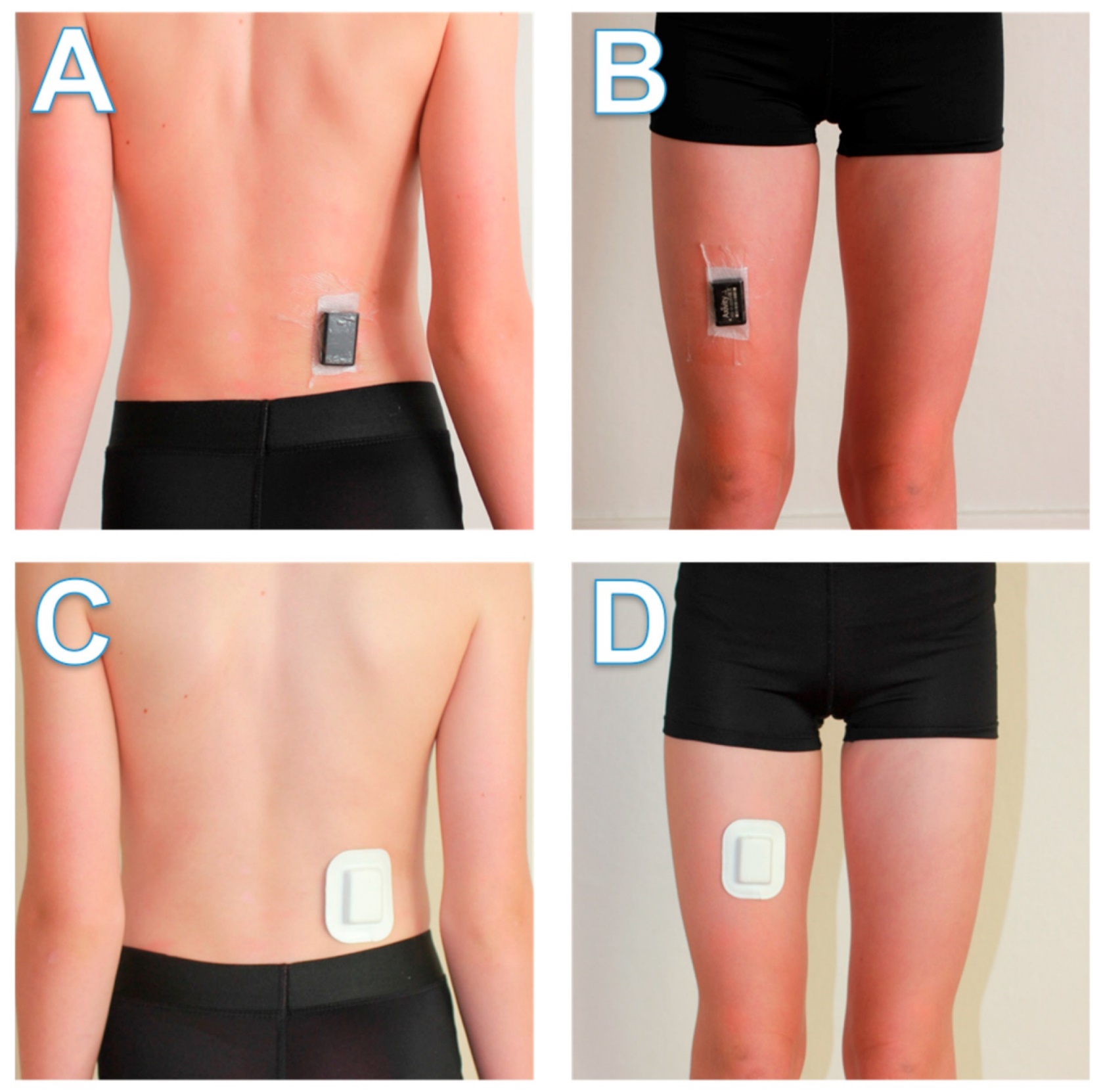


**2/3^rd^ of distance from top of right thigh to knee**

Top of right thigh

Right Knee


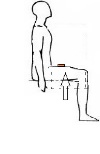
**How to wear the device**

Find where to attach the device:

1. Sit down on a chair so that your feet are flat on the ground.
2. Looking at the pictures on page 1, find the top of your right thigh and your right knee.
3. Measure 2/3^rd^ of the distance between your right thigh and knee. This is where the device will be attached onto your skin. It should be slightly closer to your knee.

Prepare your skin:

1. Carefully shave the area where the device will be attached if needed.
2. Clean the area with the alcohol wipe. Let the area dry for a few seconds.

Attach the device:

1. Stick **Tape 2** directly onto the shaved area of your thigh (see **big white tape** in Picture 1).
2. Next, stick **Tape 1** on the middle of **Tape 2** (see **smaller white tape** in Picture 1).
3. Next, stick the **device** on top of **Tape 1**. Make sure the white arrow on the device points down towards your knees (see Picture 2).
4. Next, place **Tape 3** so it covers the device completely (see **big clear tape** in Picture 3).
5. Starting from the middle, press **Tape 3** firmly down onto the device.
6. Then, slowly flatten (smooth) the tape against your skin as you move out towards the edges.
7. Try to flatten air bubbles or wrinkles if you can. This helps protect the device from water or dust.
8. Make sure the device is firmly attached to your thigh.


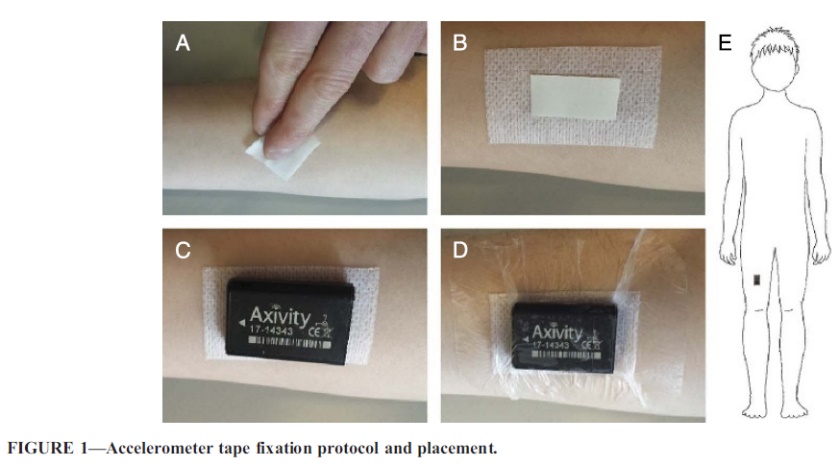


| **Picture 1** | **Picture 2** | **Picture 3** |
| --- | --- | --- |
| 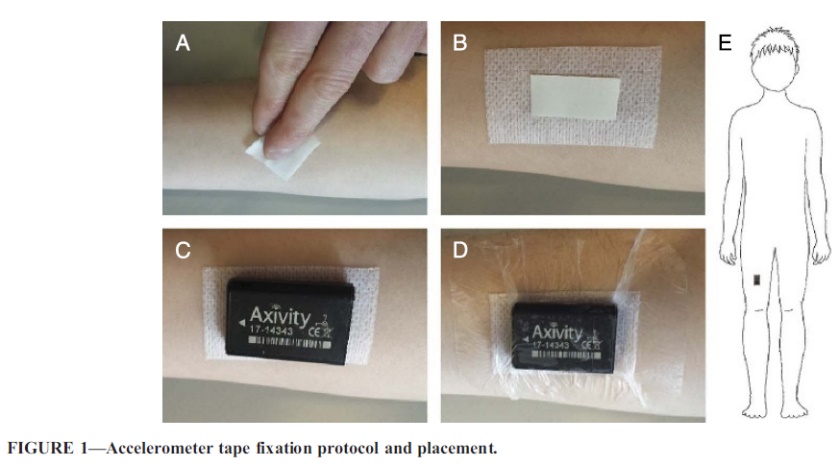 | 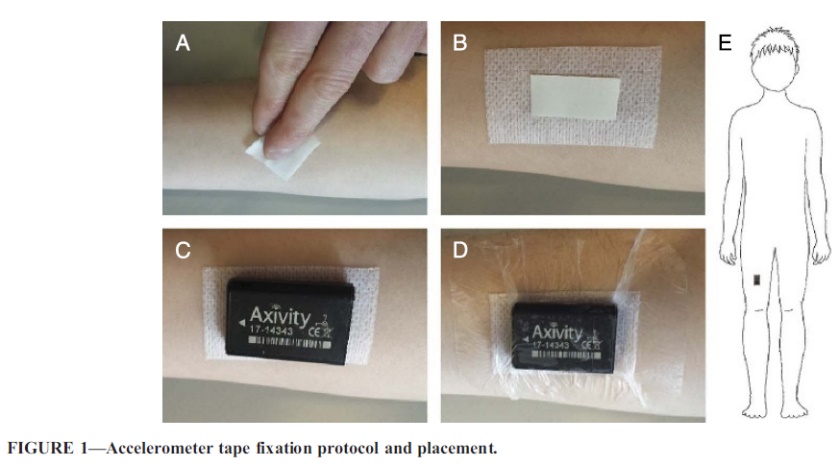 | 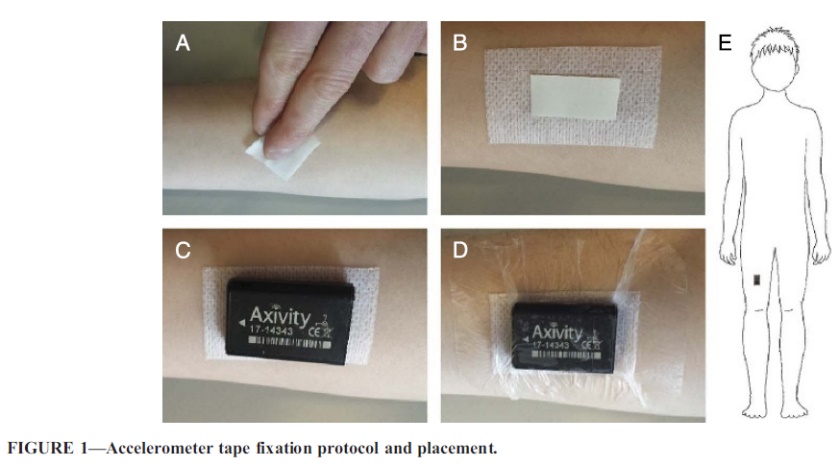 |

The **arrow** on the device should **point DOWN towards your knee**

**What if the tape peels off?**

- If the tape peels off, please remove it carefully and replace it with the spare tape provided to you. Make sure your skin and the device are clean and dry before attaching the new spare tape.

**When and how do I remove the tape and activity device?**

- You should remove the tape after wearing the activity device for 7 days. For example, if you started wearing the device on Monday morning, remove it on the following Monday morning.
- To remove the tape, hold down your skin and start by slowly peeling one edge of the tape. Continue to pull the tape gently in the direction towards your knee. Avoid peeling the tape without holding down your skin first, to prevent stretching or injuring your skin.
- For sensitive skin, apply a **small** amount of sensitive lotions or oil on the tape before trying to remove the tape. Throw away the used tape only. **Do not** throw away the activity device.

**If you experience skin irritation (e.g. redness, itchiness)**

- Skin irritations due to the tape may occur. If this happens, clearly remove the tape and device from your right leg and attach it to your left leg following the instructions on page 1. If you continue to experience irritation, remove the device completely and contact the research team.

**Returning the equipment to the research team**

- After wearing the device for 7 days, remove the device from your leg (see instructions above).

Follow the instructions (and checklist) on **page 4** of this booklet to return the device, logbook and study documents to the research team. You will receive reminders to wear and return the device.

**Participant ID: ________________ Time** (please circle)**: Baseline / 6 months**

**DAILY LOGBOOK**

**STEP 1:** **Please write the date, day and time that you put the activity device onto your right leg:**

| **DATE:** ___­__/_____/____ **DAY:** ___________________ **TIME** (HH:MM)**:** _____________ |
| --- |

**STEP 2: Every day, please write the date, time you woke up, any physical activities you completed**

**(e.g. walking, exercise), and time you went to bed/sleep.**

| **Day 1**  Date: | - Wake up time (HH:MM) *(e.g. 08:00, or 8:00am)* - Physical activity/exercise: *(e.g. walking 30 minutes, exercise program 30 minutes)* - Sleep time (HH:MM) *(e.g. 21:00, or 9:00pm)* |
| --- | --- |
| **Day 2**  Date: | - Wake up time (HH:MM) - Physical activity/exercise: - Sleep time (HH:MM) |
| **Day 3**  Date: | - Wake up time (HH:MM) - Physical activity/exercise: - Sleep time (HH:MM) |
| **Day 4**  Date: | - Wake up time (HH:MM) - Physical activity/exercise: - Sleep time (HH:MM) |
| **Day 5**  Date: | - Wake up time (HH:MM) - Physical activity/exercise: - Sleep time (HH:MM) |
| **Day 6**  Date: | - Wake up time (HH:MM) - Physical activity/exercise: - Sleep time (HH:MM) |
| **Day 7**  Date: | - Wake up time (HH:MM) - Physical activity/exercise: - Sleep time (HH:MM) |

| Participant ID: ______________________  This is your initial / 6-month assessment.  This device was sent by the research team on: ____/ _____/ ____ |
| --- |

**RETURNING ITEMS TO THE RESEARCH TEAM:**

To return the items to the research team:

1. Carefully remove the activity device, following the instructions written on page 2.
2. Carefully remove the logbook sheet (page 3 & 4) from the booklet (i.e. this page).
3. Place the items listed below into the pre-paid reply envelop given to you. The return address has already been written on the envelope.
4. Post the envelope back to the research team at your nearest post office or post box.

**CHECKLIST**: This is a checklist of the items to return to the research team

| Please return the following items: | Please tick the items you have included in this return package: |
| --- | --- |
| - Activity device |  |
| - Daily Logbook sheet (page 3 & 4) |  |
| - Weekly Diary (at **6-month only**) |  |

**QUESTIONS?**

If you are unsure how to return the equipment to the research team, please contact us:

Central Research Team

Phone: 02 9114 4808

Email: **getbacktohealthy.study@sydney.edu.au**
