# Supplementary material for: Effectiveness of a coordinated support system linking public hospitals to a health coaching service compared with usual care at discharge for patients with chronic low back pain: protocol for a randomised controlled trial
Source: BMC Musculoskelet Disord. 2021 Jul 9;22:611. doi: 10.1186/s12891-021-04479-z (PMC8272287; doi:10.1186/s12891-021-04479-z)

*
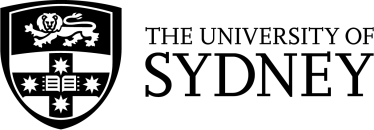
*

**Weekly Diary**


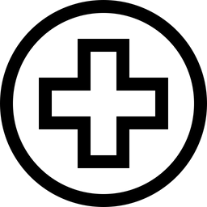


*The ‘Get Back to Healthy’ project*

Participant Initials: _________________

Participant ID: _________________­­___

Date (baseline): ___ /___ /­­___

Date (6-months): ___ /___ /­­___

**PURPOSE OF THE WEEKLY DIARY**

- Completing the weekly diary will help us monitor your safety.
- The diary will record any discomfort or incidents that may occur during the treatment period (6 months from joining the study).
- The research team will send you reminders to complete the diary.
- Please return this diary after 6 months (see page 3).

**INSTRUCTIONS: HOW TO COMPLETE AN ENTRY**

- Start from Week 1 (page 5). Please complete an entry **every week.**
- Please write the date at the start of each week.
- Tick any boxes which may apply to you (example on page 4). If no boxes apply to you, there is no need for further action.
- Examples of discomfort that often occur from participating in exercise include:
  - Flare-ups of back pain (i.e., increased back pain) that can be self-managed
  - Muscle soreness, swelling, or muscle cramps related to commencement of unaccustomed exercise
  - Trips and/or falls, that have not resulted in an injury.

These events usually resolve after a few days and are often self-managed without the need for additional medical attention.

**If a serious incident occurs:**

- - **Call 000 if it is an emergency.**
  - **Let the research team know** as soon as possible on 02 9114 4808.
  - **Record the event** in your diary entry and **email a copy of the entry** to the research team as soon as possible (detailed instructions on page 17-18 of this booklet).

**RETURNING THE DIARY**

- Please keep this diary safe and do not lose it!
- **After 6-months**, please return the diary to the research team.
- **At 6 months**, we will send a package to you containing a pre-paid reply envelope. Please use the envelope the return this diary.

**EXAMPLE ENTRY**

Below is an example of how you can complete the diary.

**Week 1:**  (Date: 13/01/2020)

| Please add a tick (✔) to as many as appropriate:  **Did you experience any of the following this week?**   \| 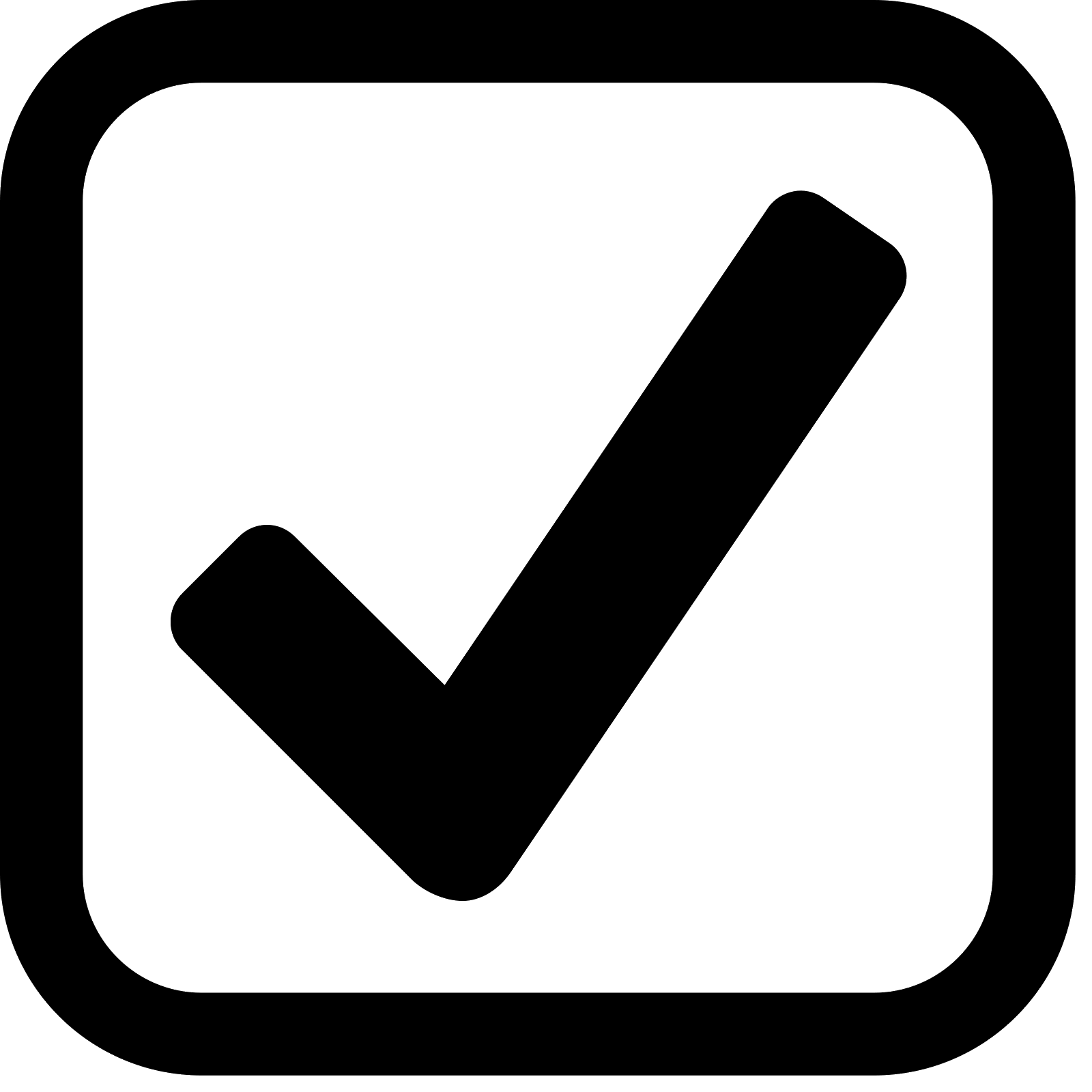 Increased back pain  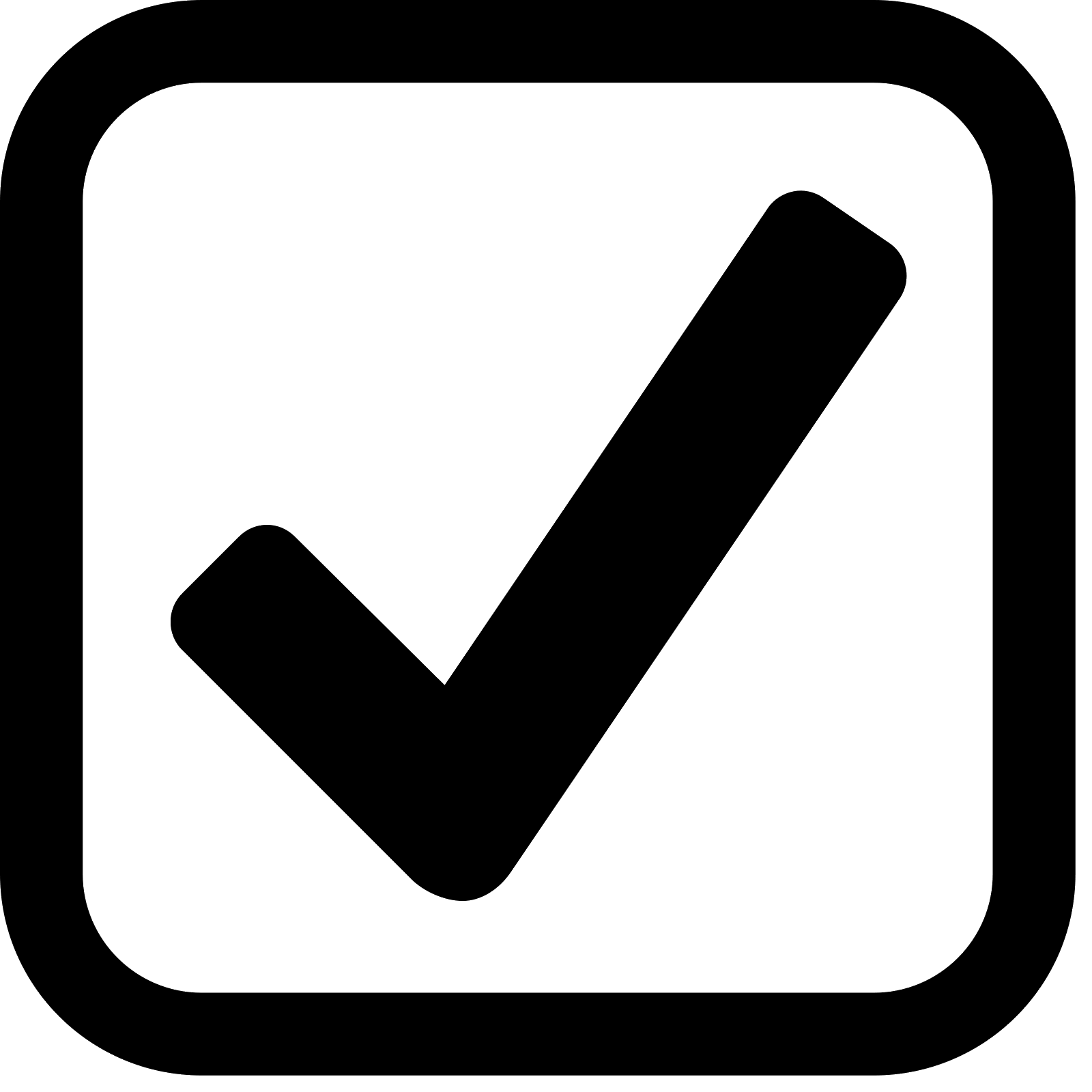 Pain elsewhere  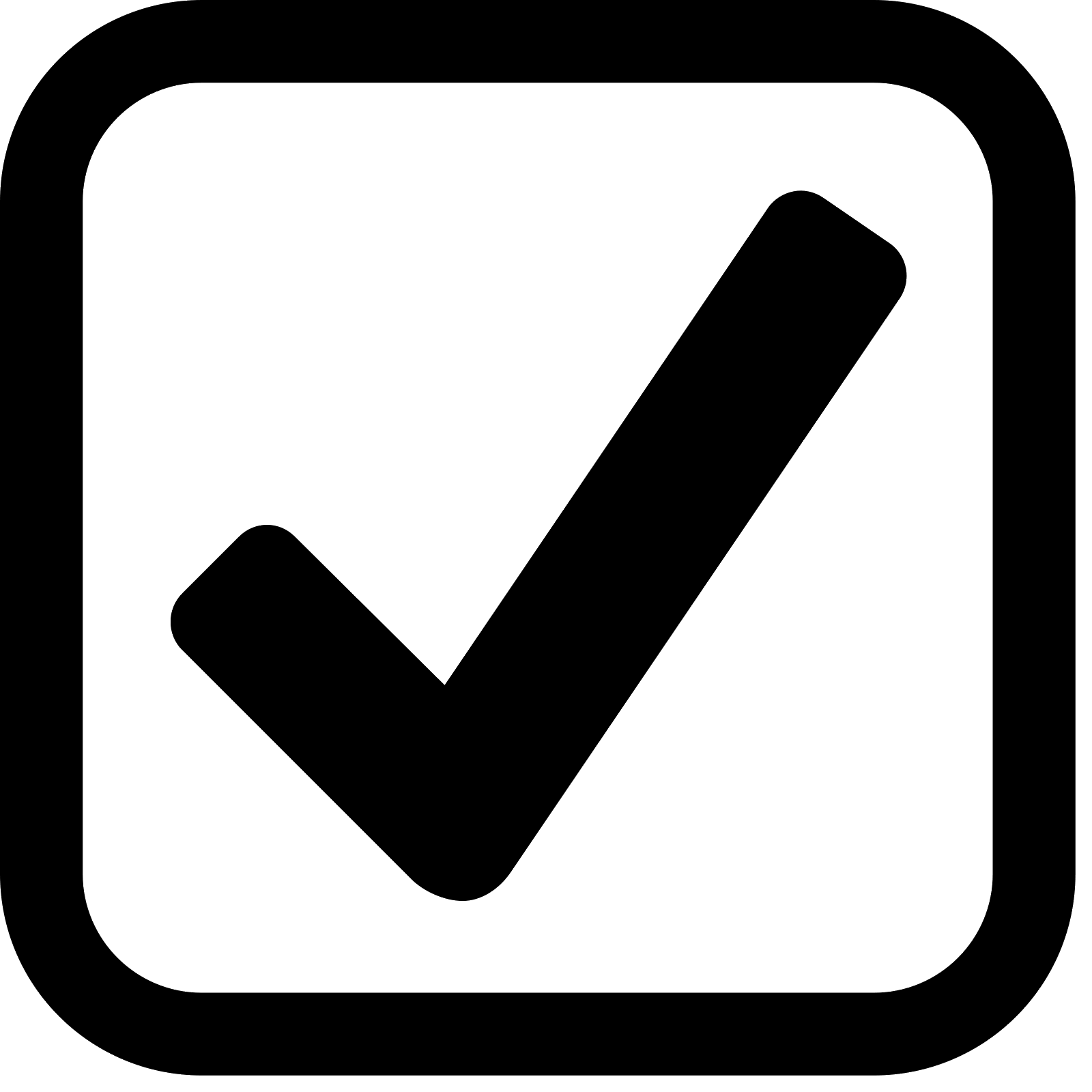 Muscle soreness  ☐ Swelling  ☐ Muscle cramp \| ☐ Trip/fall  ☐ Emotional distress  ☐ Serious event  ☐ Other symptoms, please specify________  ___________________________________________ \| \| --- \| --- \|   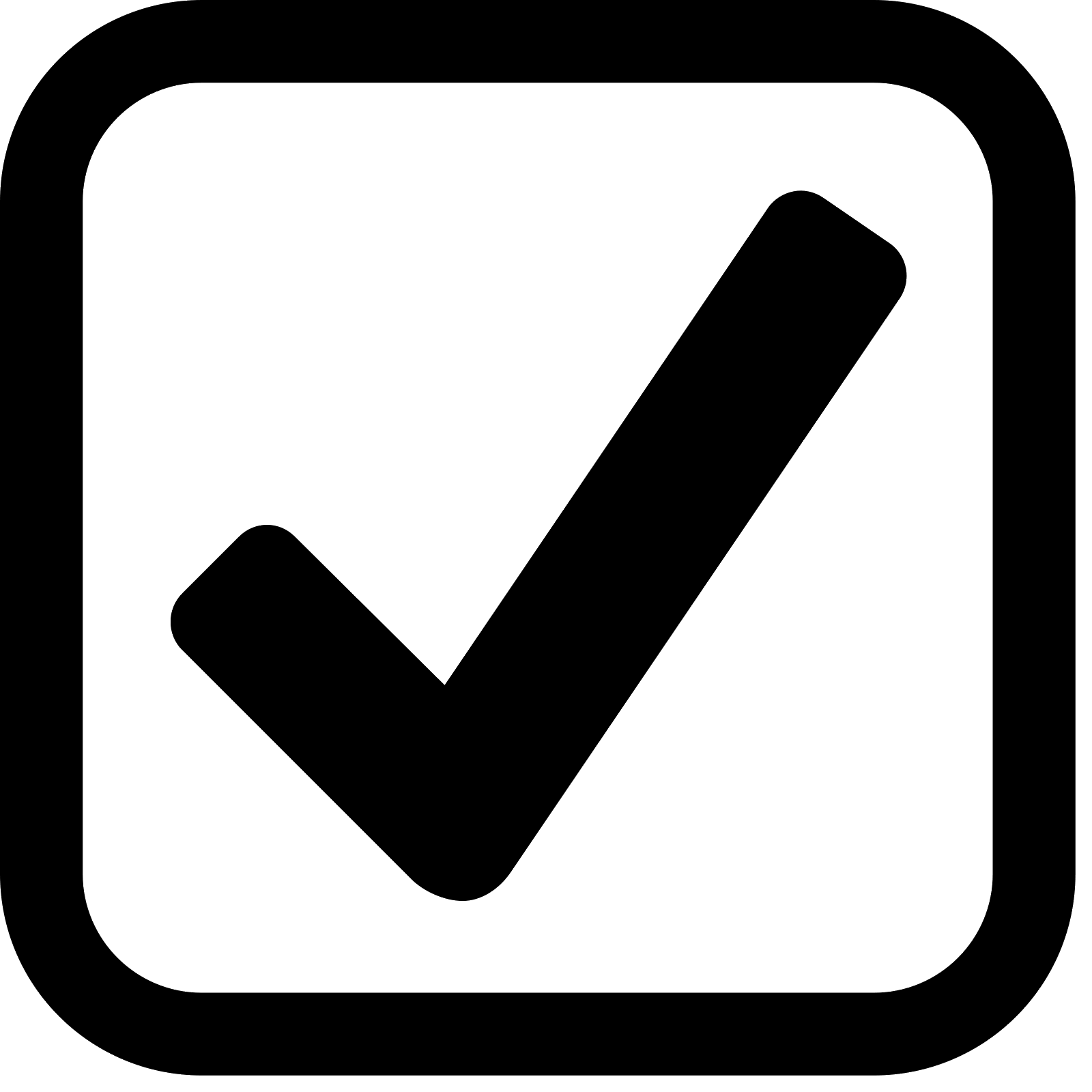**Did any of the above last more than 24 hours?**  No ☐ Yes  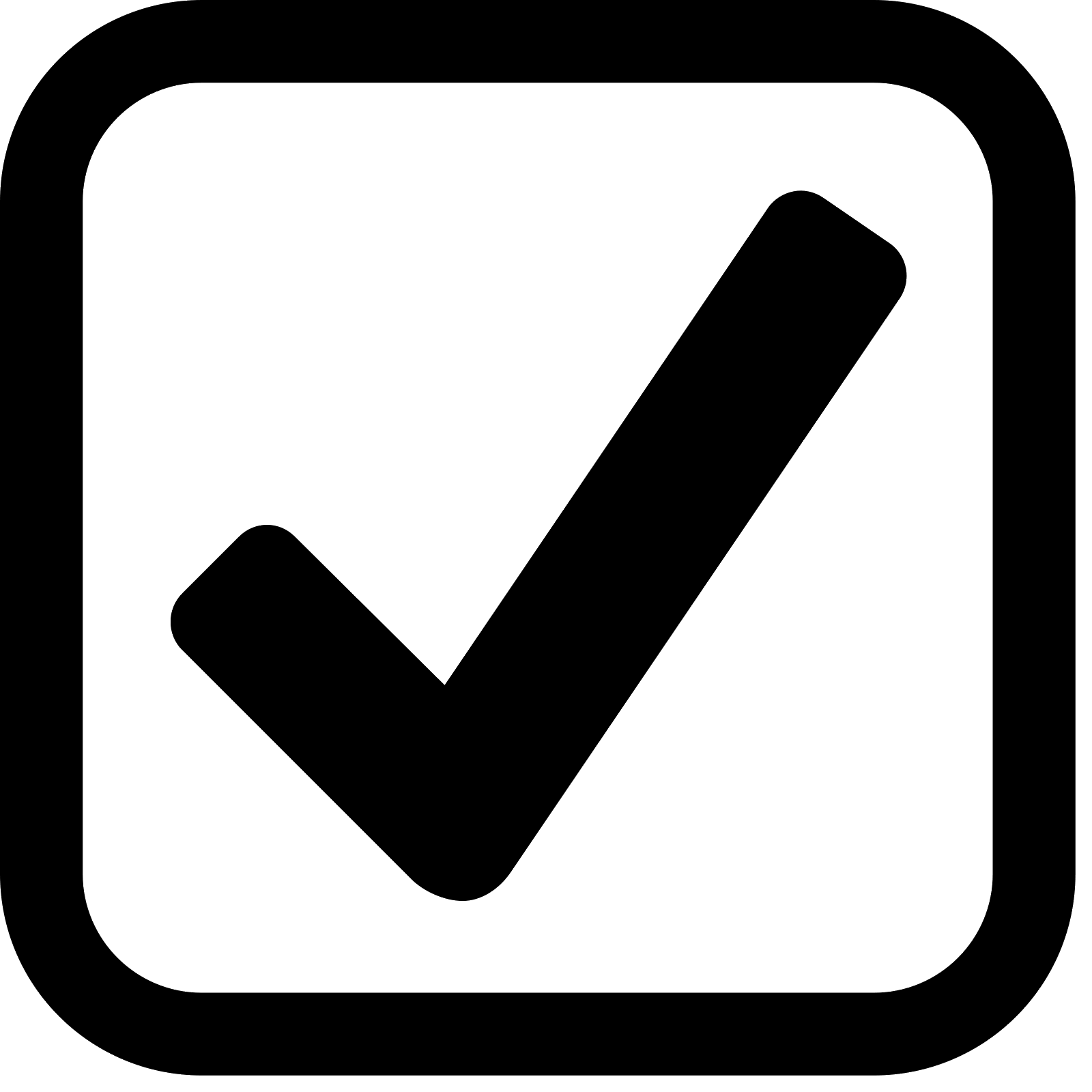**Did you require medical attention?**  No ☐ Yes |
| --- | --- | --- |

Note: It can be easy to lose track of the week number. Please write the date at the start of each week to help you keep track (e.g. each Monday).

**QUESTIONS**

- If you have concerns about your participation in the study, please **contact the central research team** at 02 9114 4808 or [**getbacktohealthy.study@sydney.edu.au**](mailto:getbacktohealthy.study@sydney.edu.au)

**Diary entries start on the next page.**

**Weekly Diary**

If you ticked **any** of the boxes below, please **email** a copy of this page to **getbacktohealthy.study@sydney.edu.au** (instructions on page 17-18).

**Week 1:** (Date / / )

| Please add a tick (✔) to as many as appropriate:  **Did you experience any of the following this week?**   \| ☐ Increased back pain  ☐ Pain elsewhere  ☐ Muscle soreness  ☐ Swelling  ☐ Muscle cramp \| ☐ Trip/fall  ☐ Emotional distress  ☐ Serious event  ☐ Other symptoms, please describe ___________________________________________ \| \| --- \| --- \|   **Did any of the above last more than 24 hours?** ☐ No ☐ Yes  **Did you require medical attention?** ☐ No ☐ Yes  If you feel that taking part in the study has caused these symptoms or you are concerned, please contact the central study team on 02 9114 4808. Call 000 if it is an emergency. |
| --- | --- | --- |

**Week 2:** (Date / / )

| Please add a tick (✔) to as many as appropriate:  **Did you experience any of the following this week?**   \| ☐ Increased back pain  ☐ Pain elsewhere  ☐ Muscle soreness  ☐ Swelling  ☐ Cramp \| ☐ Trip/fall  ☐ Emotional distress  ☐ Serious event  ☐ Other symptoms, please describe ___________________________________________ \| \| --- \| --- \|   **Did any of the above last more than 24 hours?** ☐ No ☐ Yes  **Did you require medical attention?** ☐ No ☐ Yes  If you feel that taking part in the study has caused these symptoms or you are concerned, please contact the central study team on 02 9114 4808. Call 000 if it is an emergency. |
| --- | --- | --- |

If you ticked **any** of the boxes below, please **email** a copy of this page to **getbacktohealthy.study@sydney.edu.au** (instructions on page 17-18).

**Week 3:** (Date / / )

| Please add a tick (✔) to as many as appropriate:  **Did you experience any of the following this week?**   \| ☐ Increased back pain  ☐ Pain elsewhere  ☐ Muscle soreness  ☐ Swelling  ☐ Muscle cramp \| ☐ Trip/fall  ☐ Emotional distress  ☐ Serious event  ☐ Other symptoms, please describe ___________________________________________ \| \| --- \| --- \|   **Did any of the above last more than 24 hours?** ☐ No ☐ Yes  **Did you require medical attention?** ☐ No ☐ Yes  If you feel that taking part in the study has caused these symptoms or you are concerned, please contact the central study team on 02 9114 4808. Call 000 if it is an emergency. |
| --- | --- | --- |

**Week 4:** (Date / / )

| Please add a tick (✔) to as many as appropriate:  **Did you experience any of the following this week?**   \| ☐ Increased back pain  ☐ Pain elsewhere  ☐ Muscle soreness  ☐ Swelling  ☐ Cramp \| ☐ Trip/fall  ☐ Emotional distress  ☐ Serious event  ☐ Other symptoms, please describe ___________________________________________ \| \| --- \| --- \|   **Did any of the above last more than 24 hours?** ☐ No ☐ Yes  **Did you require medical attention?** ☐ No ☐ Yes  If you feel that taking part in the study has caused these symptoms or you are concerned, please contact the central study team on 02 9114 4808. Call 000 if it is an emergency. |
| --- | --- | --- |

If you ticked **any** of the boxes below, please **email** a copy of this page to **getbacktohealthy.study@sydney.edu.au** (instructions on page 17-18).

**Week 5:** (Date / / )

| Please add a tick (✔) to as many as appropriate:  **Did you experience any of the following this week?**   \| ☐ Increased back pain  ☐ Pain elsewhere  ☐ Muscle soreness  ☐ Swelling  ☐ Cramp \| ☐ Trip/fall  ☐ Emotional distress  ☐ Serious event  ☐ Other symptoms, please describe ___________________________________________ \| \| --- \| --- \|   **Did any of the above last more than 24 hours?** ☐ No ☐ Yes  **Did you require medical attention?** ☐ No ☐ Yes  If you feel that taking part in the study has caused these symptoms or you are concerned, please contact the central study team on 02 9114 4808. Call 000 if it is an emergency. |
| --- | --- | --- |

**Week 6:** (Date / / )

| Please add a tick (✔) to as many as appropriate:  **Did you experience any of the following this week?**   \| ☐ Increased back pain  ☐ Pain elsewhere  ☐ Muscle soreness  ☐ Swelling  ☐ Cramp \| ☐ Trip/fall  ☐ Emotional distress  ☐ Serious event  ☐ Other symptoms, please describe ___________________________________________ \| \| --- \| --- \|   **Did any of the above last more than 24 hours?** ☐ No ☐ Yes  **Did you require medical attention?** ☐ No ☐ Yes  If you feel that taking part in the study has caused these symptoms or you are concerned, please contact the central study team on 02 9114 4808. Call 000 if it is an emergency. |
| --- | --- | --- |

If you ticked **any** of the boxes below, please **email** a copy of this page to **getbacktohealthy.study@sydney.edu.au** (instructions on page 17-18).

**Week 7:** (Date / / )

| Please add a tick (✔) to as many as appropriate:  **Did you experience any of the following this week?**   \| ☐ Increased back pain  ☐ Pain elsewhere  ☐ Muscle soreness  ☐ Swelling  ☐ Cramp \| ☐ Trip/fall  ☐ Emotional distress  ☐ Serious event  ☐ Other symptoms, please describe ___________________________________________ \| \| --- \| --- \|   **Did any of the above last more than 24 hours?** ☐ No ☐ Yes  **Did you require medical attention?** ☐ No ☐ Yes  If you feel that taking part in the study has caused these symptoms or you are concerned, please contact the central study team on 02 9114 4808. Call 000 if it is an emergency. |
| --- | --- | --- |

**Week 8:** (Date / / )

| Please add a tick (✔) to as many as appropriate:  **Did you experience any of the following this week?**   \| ☐ Increased back pain  ☐ Pain elsewhere  ☐ Muscle soreness  ☐ Swelling  ☐ Cramp \| ☐ Trip/fall  ☐ Emotional distress  ☐ Serious event  ☐ Other symptoms, please describe ___________________________________________ \| \| --- \| --- \|   **Did any of the above last more than 24 hours?** ☐ No ☐ Yes  **Did you require medical attention?** ☐ No ☐ Yes  If you feel that taking part in the study has caused these symptoms or you are concerned, please contact the central study team on 02 9114 4808. Call 000 if it is an emergency. |
| --- | --- | --- |

If you ticked **any** of the boxes below, please **email** a copy of this page to **getbacktohealthy.study@sydney.edu.au** (instructions on page 17-18).

**Week 9:** (Date / / )

| Please add a tick (✔) to as many as appropriate:  **Did you experience any of the following this week?**   \| ☐ Increased back pain  ☐ Pain elsewhere  ☐ Muscle soreness  ☐ Swelling  ☐ Cramp \| ☐ Trip/fall  ☐ Emotional distress  ☐ Serious event  ☐ Other symptoms, please describe ___________________________________________ \| \| --- \| --- \|   **Did any of the above last more than 24 hours?** ☐ No ☐ Yes  **Did you require medical attention?** ☐ No ☐ Yes  If you feel that taking part in the study has caused these symptoms or you are concerned, please contact the central study team on 02 9114 4808. Call 000 if it is an emergency. |
| --- | --- | --- |

**Week 10:** (Date / / )

| Please add a tick (✔) to as many as appropriate:  **Did you experience any of the following this week?**   \| ☐ Increased back pain  ☐ Pain elsewhere  ☐ Muscle soreness  ☐ Swelling  ☐ Cramp \| ☐ Trip/fall  ☐ Emotional distress  ☐ Serious event  ☐ Other symptoms, please describe ___________________________________________ \| \| --- \| --- \|   **Did any of the above last more than 24 hours?** ☐ No ☐ Yes  **Did you require medical attention?** ☐ No ☐ Yes  If you feel that taking part in the study has caused these symptoms or you are concerned, please contact the central study team on 02 9114 4808. Call 000 if it is an emergency. |
| --- | --- | --- |

If you ticked **any** of the boxes below, please **email** a copy of this page to **getbacktohealthy.study@sydney.edu.au** (instructions on page 17-18).

**Week 11:** (Date / / )

| Please add a tick (✔) to as many as appropriate:  **Did you experience any of the following this week?**   \| ☐ Increased back pain  ☐ Pain elsewhere  ☐ Muscle soreness  ☐ Swelling  ☐ Cramp \| ☐ Trip/fall  ☐ Emotional distress  ☐ Serious event  ☐ Other symptoms, please describe ___________________________________________ \| \| --- \| --- \|   **Did any of the above last more than 24 hours?** ☐ No ☐ Yes  **Did you require medical attention?** ☐ No ☐ Yes  If you feel that taking part in the study has caused these symptoms or you are concerned, please contact the central study team on 02 9114 4808. Call 000 if it is an emergency. |
| --- | --- | --- |

**Week 12:** (Date / / )

| Please add a tick (✔) to as many as appropriate:  **Did you experience any of the following this week?**   \| ☐ Increased back pain  ☐ Pain elsewhere  ☐ Muscle soreness  ☐ Swelling  ☐ Cramp \| ☐ Trip/fall  ☐ Emotional distress  ☐ Serious event  ☐ Other symptoms, please describe ___________________________________________ \| \| --- \| --- \|   **Did any of the above last more than 24 hours?** ☐ No ☐ Yes  **Did you require medical attention?** ☐ No ☐ Yes  If you feel that taking part in the study has caused these symptoms or you are concerned, please contact the central study team on 02 9114 4808. Call 000 if it is an emergency. |
| --- | --- | --- |

If you ticked **any** of the boxes below, please **email** a copy of this page to **getbacktohealthy.study@sydney.edu.au** (instructions on page 17-18).

**Week 13:** (Date / / )

| Please add a tick (✔) to as many as appropriate:  **Did you experience any of the following this week?**   \| ☐ Increased back pain  ☐ Pain elsewhere  ☐ Muscle soreness  ☐ Swelling  ☐ Cramp \| ☐ Trip/fall  ☐ Emotional distress  ☐ Serious event  ☐ Other symptoms, please describe ___________________________________________ \| \| --- \| --- \|   **Did any of the above last more than 24 hours?** ☐ No ☐ Yes  **Did you require medical attention?** ☐ No ☐ Yes  If you feel that taking part in the study has caused these symptoms or you are concerned, please contact the central study team on 02 9114 4808. Call 000 if it is an emergency. |
| --- | --- | --- |

**Week 14:** (Date / / )

| Please add a tick (✔) to as many as appropriate:  **Did you experience any of the following this week?**   \| ☐ Increased back pain  ☐ Pain elsewhere  ☐ Muscle soreness  ☐ Swelling  ☐ Cramp \| ☐ Trip/fall  ☐ Emotional distress  ☐ Serious event  ☐ Other symptoms, please describe ___________________________________________ \| \| --- \| --- \|   **Did any of the above last more than 24 hours?** ☐ No ☐ Yes  **Did you require medical attention?** ☐ No ☐ Yes  If you feel that taking part in the study has caused these symptoms or you are concerned, please contact the central study team on 02 9114 4808. Call 000 if it is an emergency. |
| --- | --- | --- |

If you ticked **any** of the boxes below, please **email** a copy of this page to **getbacktohealthy.study@sydney.edu.au** (instructions on page 17-18).

**Week 15:** (Date / / )

| Please add a tick (✔) to as many as appropriate:  **Did you experience any of the following this week?**   \| ☐ Increased back pain  ☐ Pain elsewhere  ☐ Muscle soreness  ☐ Swelling  ☐ Cramp \| ☐ Trip/fall  ☐ Emotional distress  ☐ Serious event  ☐ Other symptoms, please describe ___________________________________________ \| \| --- \| --- \|   **Did any of the above last more than 24 hours?** ☐ No ☐ Yes  **Did you require medical attention?** ☐ No ☐ Yes  If you feel that taking part in the study has caused these symptoms or you are concerned, please contact the central study team on 02 9114 4808. Call 000 if it is an emergency. |
| --- | --- | --- |

**Week 16:** (Date / / )

| Please add a tick (✔) to as many as appropriate:  **Did you experience any of the following this week?**   \| ☐ Increased back pain  ☐ Pain elsewhere  ☐ Muscle soreness  ☐ Swelling  ☐ Cramp \| ☐ Trip/fall  ☐ Emotional distress  ☐ Serious event  ☐ Other symptoms, please describe ___________________________________________ \| \| --- \| --- \|   **Did any of the above last more than 24 hours?** ☐ No ☐ Yes  **Did you require medical attention?** ☐ No ☐ Yes  If you feel that taking part in the study has caused these symptoms or you are concerned, please contact the central study team on 02 9114 4808. Call 000 if it is an emergency. |
| --- | --- | --- |

If you ticked **any** of the boxes below, please **email** a copy of this page to **getbacktohealthy.study@sydney.edu.au** (instructions on page 17-18).

**Week 17:** (Date / / )

| Please add a tick (✔) to as many as appropriate:  **Did you experience any of the following this week?**   \| ☐ Increased back pain  ☐ Pain elsewhere  ☐ Muscle soreness  ☐ Swelling  ☐ Cramp \| ☐ Trip/fall  ☐ Emotional distress  ☐ Serious event  ☐ Other symptoms, please describe ___________________________________________ \| \| --- \| --- \|   **Did any of the above last more than 24 hours?** ☐ No ☐ Yes  **Did you require medical attention?** ☐ No ☐ Yes  If you feel that taking part in the study has caused these symptoms or you are concerned, please contact the central study team on 02 9114 4808. Call 000 if it is an emergency. |
| --- | --- | --- |

**Week 18:** (Date / / )

| Please add a tick (✔) to as many as appropriate:  **Did you experience any of the following this week?**   \| ☐ Increased back pain  ☐ Pain elsewhere  ☐ Muscle soreness  ☐ Swelling  ☐ Cramp \| ☐ Trip/fall  ☐ Emotional distress  ☐ Serious event  ☐ Other symptoms, please describe ___________________________________________ \| \| --- \| --- \|   **Did any of the above last more than 24 hours?** ☐ No ☐ Yes  **Did you require medical attention?** ☐ No ☐ Yes  If you feel that taking part in the study has caused these symptoms or you are concerned, please contact the central study team on 02 9114 4808. Call 000 if it is an emergency. |
| --- | --- | --- |

If you ticked **any** of the boxes below, please **email** a copy of this page to **getbacktohealthy.study@sydney.edu.au** (instructions on page 17-18).

**Week 19:** (Date / / )

| Please add a tick (✔) to as many as appropriate:  **Did you experience any of the following this week?**   \| ☐ Increased back pain  ☐ Pain elsewhere  ☐ Muscle soreness  ☐ Swelling  ☐ Cramp \| ☐ Trip/fall  ☐ Emotional distress  ☐ Serious event  ☐ Other symptoms, please describe ___________________________________________ \| \| --- \| --- \|   **Did any of the above last more than 24 hours?** ☐ No ☐ Yes  **Did you require medical attention?** ☐ No ☐ Yes  If you feel that taking part in the study has caused these symptoms or you are concerned, please contact the central study team on 02 9114 4808. Call 000 if it is an emergency. |
| --- | --- | --- |

**Week 20:** (Date / / )

| Please add a tick (✔) to as many as appropriate:  **Did you experience any of the following this week?**   \| ☐ Increased back pain  ☐ Pain elsewhere  ☐ Muscle soreness  ☐ Swelling  ☐ Cramp \| ☐ Trip/fall  ☐ Emotional distress  ☐ Serious event  ☐ Other symptoms, please describe ___________________________________________ \| \| --- \| --- \|   **Did any of the above last more than 24 hours?** ☐ No ☐ Yes  **Did you require medical attention?** ☐ No ☐ Yes  If you feel that taking part in the study has caused these symptoms or you are concerned, please contact the central study team on 02 9114 4808. Call 000 if it is an emergency. |
| --- | --- | --- |

If you ticked **any** of the boxes below, please **email** a copy of this page to **getbacktohealthy.study@sydney.edu.au** (instructions on page 17-18).

**Week 21:** (Date / / )

| Please add a tick (✔) to as many as appropriate:  **Did you experience any of the following this week?**   \| ☐ Increased back pain  ☐ Pain elsewhere  ☐ Muscle soreness  ☐ Swelling  ☐ Cramp \| ☐ Trip/fall  ☐ Emotional distress  ☐ Serious event  ☐ Other symptoms, please describe ___________________________________________ \| \| --- \| --- \|   **Did any of the above last more than 24 hours?** ☐ No ☐ Yes  **Did you require medical attention?** ☐ No ☐ Yes  If you feel that taking part in the study has caused these symptoms or you are concerned, please contact the central study team on 02 9114 4808. Call 000 if it is an emergency. |
| --- | --- | --- |

**Week 22:** (Date / / )

| Please add a tick (✔) to as many as appropriate:  **Did you experience any of the following this week?**   \| ☐ Increased back pain  ☐ Pain elsewhere  ☐ Muscle soreness  ☐ Swelling  ☐ Cramp \| ☐ Trip/fall  ☐ Emotional distress  ☐ Serious event  ☐ Other symptoms, please describe ___________________________________________ \| \| --- \| --- \|   **Did any of the above last more than 24 hours?** ☐ No ☐ Yes  **Did you require medical attention?** ☐ No ☐ Yes  If you feel that taking part in the study has caused these symptoms or you are concerned, please contact the central study team on 02 9114 4808. Call 000 if it is an emergency. |
| --- | --- | --- |

If you ticked **any** of the boxes below, please **email** a copy of this page to **getbacktohealthy.study@sydney.edu.au** (instructions on page 17-18).

**Week 23:** (Date / / )

| Please add a tick (✔) to as many as appropriate:  **Did you experience any of the following this week?**   \| ☐ Increased back pain  ☐ Pain elsewhere  ☐ Muscle soreness  ☐ Swelling  ☐ Cramp \| ☐ Trip/fall  ☐ Emotional distress  ☐ Serious event  ☐ Other symptoms, please describe ___________________________________________ \| \| --- \| --- \|   **Did any of the above last more than 24 hours?** ☐ No ☐ Yes  **Did you require medical attention?** ☐ No ☐ Yes  If you feel that taking part in the study has caused these symptoms or you are concerned, please contact the central study team on 02 9114 4808. Call 000 if it is an emergency. |
| --- | --- | --- |

**Week 24:** (Date / / )

| Please add a tick (✔) to as many as appropriate:  **Did you experience any of the following this week?**   \| ☐ Increased back pain  ☐ Pain elsewhere  ☐ Muscle soreness  ☐ Swelling  ☐ Cramp \| ☐ Trip/fall  ☐ Emotional distress  ☐ Serious event  ☐ Other symptoms, please describe ___________________________________________ \| \| --- \| --- \|   **Did any of the above last more than 24 hours?** ☐ No ☐ Yes  **Did you require medical attention?** ☐ No ☐ Yes  If you feel that taking part in the study has caused these symptoms or you are concerned, please contact the central study team on 02 9114 4808. Call 000 if it is an emergency. |
| --- | --- | --- |

If you ticked **any** of the boxes below, please **email** a copy of this page to **getbacktohealthy.study@sydney.edu.au** (instructions on page 17-18).

**Week 25:** (Date / / )

| Please add a tick (✔) to as many as appropriate:  **Did you experience any of the following this week?**   \| ☐ Increased back pain  ☐ Pain elsewhere  ☐ Muscle soreness  ☐ Swelling  ☐ Cramp \| ☐ Trip/fall  ☐ Emotional distress  ☐ Serious event  ☐ Other symptoms, please describe ___________________________________________ \| \| --- \| --- \|   **Did any of the above last more than 24 hours?** ☐ No ☐ Yes  **Did you require medical attention?** ☐ No ☐ Yes  If you feel that taking part in the study has caused these symptoms or you are concerned, please contact the central study team on 02 9114 4808. Call 000 if it is an emergency. |
| --- | --- | --- |

**Week 26:** (Date / / )

| Please add a tick (✔) to as many as appropriate:  **Did you experience any of the following this week?**   \| ☐ Increased back pain  ☐ Pain elsewhere  ☐ Muscle soreness  ☐ Swelling  ☐ Cramp \| ☐ Trip/fall  ☐ Emotional distress  ☐ Serious event  ☐ Other symptoms, please describe ___________________________________________ \| \| --- \| --- \|   **Did any of the above last more than 24 hours?** ☐ No ☐ Yes  **Did you require medical attention?** ☐ No ☐ Yes  If you feel that taking part in the study has caused these symptoms or you are concerned, please contact the central study team on 02 9114 4808. Call 000 if it is an emergency. |
| --- | --- | --- |

**Instructions for sending a diary entry via email:**

1. If discomfort or an incident has occurred, please complete a detailed diary entry for that week.
2. Write **your participant ID number** at the top of the page (your ID number can be found on the front of this booklet).
3. Open the camera application on your phone/tablet.
4. Take a clear picture of the whole page.
5. On your phone/tablet, open your mail account (Gmail, Outlook, Mail etc.)
6. Click on compose an email (this could be the ‘ **+** ’ symbol on your screen)
7. ‘**To’**: **getbacktohealthy.study@sydney.edu.au**
8. ‘**Subject’**: Write your participant ID number, and the week number of the entry you are sending us. *Example: Participant 001-001, Diary Week 1*
9. If you use Android: Tap on the button attach a file (paper clip symbol).
   1. Click “Attach File”.
   2. The" Mail" app will redirect you to your Photos/Files.
   3. Tap the picture of the diary page that you just took.
10. If you use iPhone: Double-tap the text field of the email message.
    1. The stripe with extra options appears. Tap the little triangle on the right of the stripe to invoke extra options.
    2. Tap "Insert Photo or Video".
    3. The" Mail" app will redirect you to your Photos.
    4. Tap the album containing the picture you just took. Then, tap the picture of the diary page that you just took.
11. You will return to your unfinished email. Tap **"Send".**

***If you are unsure, please contact the research team if you need any help***

| **Android** |
| --- |
| Participant ID number, Diary Week {number} 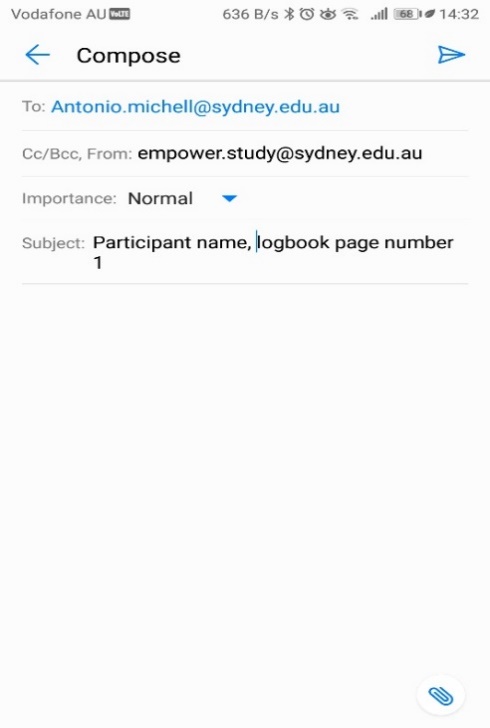 getbacktohealthy.study@sydney.edu.au |
| **iPhone** |
| getbacktohealthy.study@sydney.edu.au 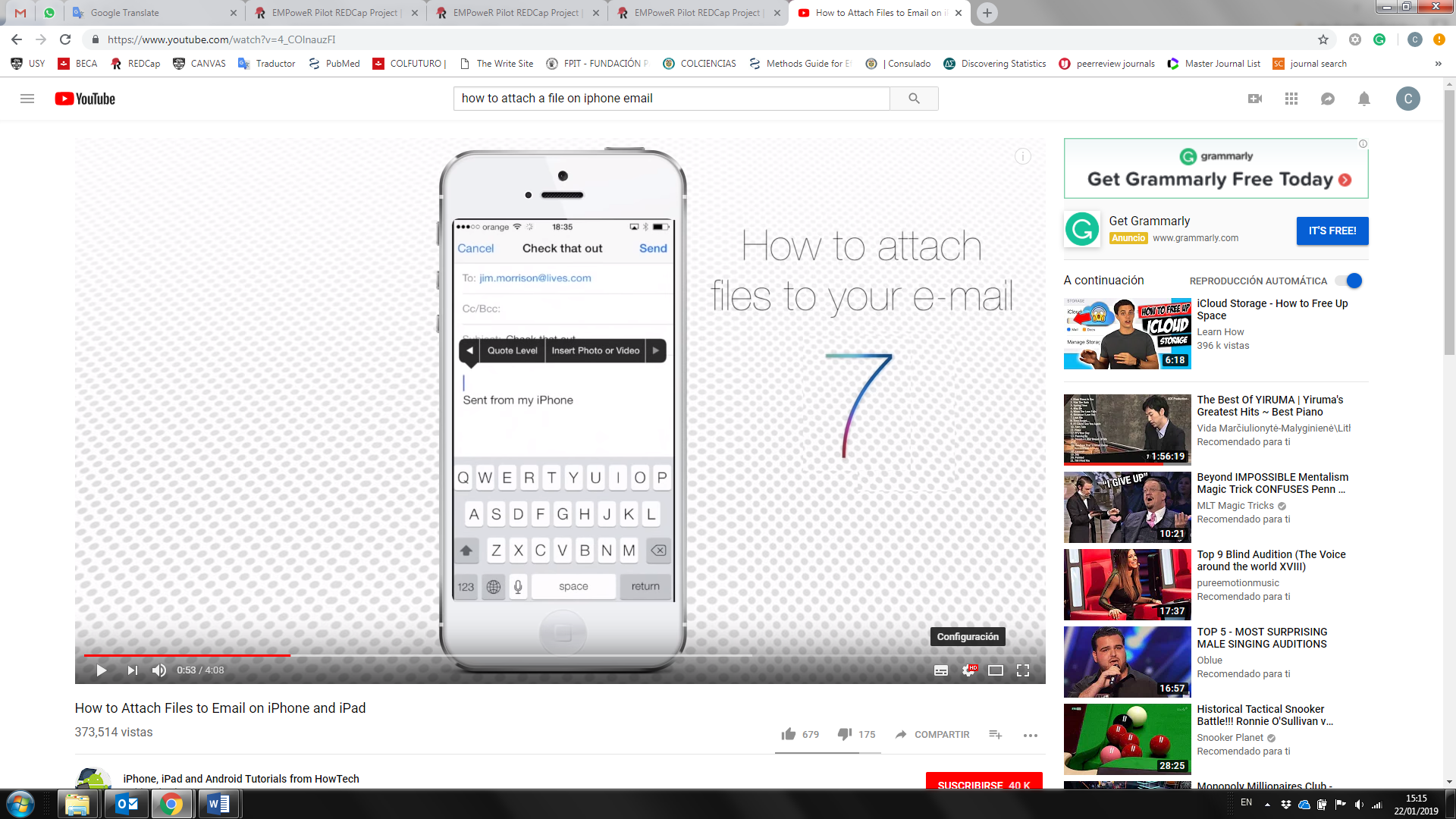 |

**Thank you for completing the weekly diary!**


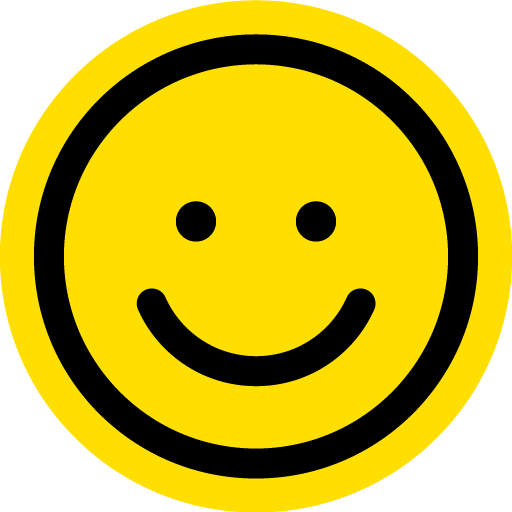

Supplement: Supplementary file 8 — Additional file 8. [file 12891_2021_4479_MOESM8_ESM.docx]
