# Supplementary material for: Effectiveness of a coordinated support system linking public hospitals to a health coaching service compared with usual care at discharge for patients with chronic low back pain: protocol for a randomised controlled trial
Source: BMC Musculoskelet Disord. 2021 Jul 9;22:611. doi: 10.1186/s12891-021-04479-z (PMC8272287; doi:10.1186/s12891-021-04479-z)
Supplement: Supplementary file 9 — Additional file 9. [file 12891_2021_4479_MOESM9_ESM.docx]

**Clinical Escalation Pathway for Health Coaches**

Indicators for Clinical Escalation

Red Flags for LBP requiring clinical escalation:

- Major trauma, minor trauma in elderly or osteo-porotic patient
- Fever, chills, night sweats, unexplained weight loss, immuno-compromised
- Night pain, non-mechanical pain, unremitting pain even at rest
- Severe or progressive sensory alteration or weakness
- Bladder or bowel dysfunction
- History of Cancer
- Intravenous drug use, steroid use

Procedures for Clinical Escalation

If a health coach identifies any red flags requiring clinical escalation during the health coaching sessions, the health coach will follow Get Healthy Service’s® Clinical Escalation Policy according to whether urgent emergency response or urgent medical review is required. If emergency response is necessitated, this may involve the health coach completing a warm transfer to 000 (Australian national emergency phone number). If urgent medical review is required, this may involve the health coach contacting the participant’s medical practitioner, as appropriate. In addition, the health coach will also prompt the participant to complete their weekly diary and contact the research team as soon as possible. In accordance with standard Get Healthy Service® procedures, the health coach may re-screen participants prior to continuing further health coaching sessions in the event clinical escalation is required. If there are any concerns, the Get Healthy Service® may contact the study team. If a participant is discharged from the Service (i.e., the study intervention), the study team will be notified of the participant’s withdrawal from the intervention.

If an adverse event occurs, other than red flag indicators or events warranting emergency response or urgent medical review, the health coach will prompt the participant to complete their weekly diary and contact the research team as soon as possible. The research team will follow-up the adverse event until resolution.
